# Comparative efficacy and safety of anti-osteoporotic therapies for kidney transplant recipients: a systematic review and network meta-analysis

**DOI:** 10.3389/fendo.2025.1689233

**Published:** 2025-11-03

**Authors:** Xiaopei Liu, Xingyao Li, Yanhong Zhao, Qi Gao, Yuan Xue, Zhongheng Wu, Xingmin Shi, Xili Wu

**Affiliations:** ^1^ Department of Integrated Traditional Chinese and Western Medicine, Second Affiliated Hospital of Xi’an Jiaotong University, Xi’an, Shaanxi, China; ^2^ Key Laboratory for Disease Prevention and Control and Health Promotion of Shaanxi Province, School of Public Health, Medical Science Center, Xi’an Jiaotong University, Xi’an, China

**Keywords:** antiosteoporosis drugs, bone mineral density, kidney transplant recipients, osteoporosis, systematic review

## Abstract

**Background:**

Kidney transplant recipients (KTRs) are at an increased risk of osteoporosis, which negatively impacts their quality of life and transplant outcomes. However, the efficacy and safety of anti-osteoporosis treatments in this group remain uncertain.

**Methods:**

We conducted a systematic search of PubMed, Embase, Web of Science, and the Cochrane Central Register of Controlled Trials up to August 1, 2024. Randomized controlled trials (RCTs) examining anti-osteoporotic medications in KTRs were included. Primary outcomes were changes in bone mineral density (BMD) at femoral neck and lumbar spine, and adverse events. We performed a frequentist network meta-analysis using random-effects models. Evidence certainty was assessed using the GRADE approach.

**Results:**

Twenty-one RCTs involving 1,066 participants were included, published between 2000 and 2021. For femoral neck BMD, bisphosphonates significantly improved BMD compared to control (MD = 0.04, 95%CI=0.00-0.09, p<0.05) based on low certainty evidence, while calcitonin was significantly superior to calcium (MD=-0.14, 95%CI=-0.28 to -0.01). Most other comparisons showed no statistically significant differences based on very low to moderate certainty evidence. For lumbar spine BMD, bisphosphonates, calcitonin, and calcium demonstrated statistically significant inferiority compared to denosumab, with bisphosphonates showing MD=-4.98 (95%CI=-6.84 to -3.13), calcitonin showing MD=-4.35 (95%CI=-6.24 to -2.47), and calcium showing MD=-5.85 (95%CI=-7.72 to -3.98), while denosumab was superior to control (MD = 5.10, 95%CI=3.25-6.95), based on low to very low certainty evidence from one RCT. Calcitonin was also significantly superior to calcium (MD = 0.60, 95%CI=0.07-1.12). For safety outcomes, no statistically significant differences were observed between interventions based on low to moderate certainty evidence.

**Conclusion:**

Denosumab appears most effective for improving lumbar spine BMD in KTRs, while calcitonin shows promise for femoral neck BMD improvement. However, the low to moderate certainty of evidence necessitates individualized treatment approaches considering patient-specific factors including renal function and safety profiles. These findings suggest current guidelines emphasizing bisphosphonates as first-line therapy may require revision, though larger long-term studies with fracture endpoints are needed to confirm these results.

**Systematic review registration:**

https://www.crd.york.ac.uk/prospero/?utm_source=chatgpt.com, identifier PROSPERO CRD42024587203.

## 1 Introduction

In patients with end-stage renal disease (ESRD), kidney transplantation has become the preferred therapy, leading to substantial improvements in both survival rates and quality of life. Indeed, kidney transplant recipients (KTRs) encounter a multitude of post-transplant complications, with mineral metabolism disorders and skeletal diseases being especially notable (1–3). Osteoporosis exhibits a markedly elevated prevalence in the KTR population, characterized by a significant reduction in bone mineral density (BMD) and a concomitant increase in fracture risk (4, 5).The skeletal complications not only have a detrimental effect on the daily lives of patients but also contribute to significant long-term medical costs, so compromising the overall effectiveness of kidney transplantation (6, 7).

Anti-osteoporosis therapy plays an important role in mitigating and preventing skeletal diseases in KTRs. Clinicians widely employ various pharmacological interventions, including bisphosphonates, vitamin D and calcium supplements, bone formation promoters, and hormonal agents, to improve BMD and reduce fracture risk in KTRs (8–10). However, the unique pathophysiological characteristics of KTRs—such as ongoing immunosuppressive therapy, variable renal function recovery, and comorbidities—may significantly affect the efficacy and safety profiles of these medications compared to the general population (3, 11–13).

Previous systematic reviews and meta-analyses of anti-osteoporosis treatments have predominantly focused on the general population. The evidence on KTRs remains unclear (14). Moreover, direct head-to-head comparisons between different anti-osteoporotic interventions in KTRs are notably scarce, with most existing studies comparing single active treatments against placebo or standard care rather than against each other (15, 16). Thus, this study aimed to comprehensively evaluate the effectiveness and safety of various anti-osteoporosis medications in KTRs through systematic review and network meta-analysis (NMA) (1–3).We compared their relative efficacy in improving BMD and reducing fracture risk, while assessing safety profiles. Our goal was to provide clinicians with robust, evidence-based guidance for selecting optimal anti-osteoporosis treatments in KTR populations (4–6).

## 2 Methods

We conducted this systematic review and network meta-analysis in accordance with the Preferred Reporting Items for Systematic Reviews and Meta-Analyses extension for Network Meta-Analyses (PRISMA-NMA) guidelines (17).The protocol was prospectively registered in PROSPERO (CRD42024587203).

### 2.1 Eligibility criteria

We included randomized controlled trials (RCTs) investigating pharmacological interventions for osteoporosis in KTRs. Eligible studies enrolled participants of any gender, at any time post-transplantation, and with end-stage renal disease of any etiology leading to transplantation. We considered interventions including any pharmacological agent for the prevention or treatment of osteoporosis, including but not limited to bisphosphonates, selective estrogen receptor modulators, parathyroid hormone and analogues, anti-RANKL monoclonal antibodies, calcium and vitamin D supplementation, hormone replacement therapy, and novel anti-osteoporotic agents. Comparators could be placebo, standard care (defined as no specific anti-osteoporotic treatment), or other active pharmacological interventions, allowing for head-to-head comparisons. The outcomes of interest were changes in BMD and the incidence of adverse events.

We imposed no restrictions on the duration of follow-up or publication date. We excluded studies involving combined organ transplant recipients, non-English publications, non-randomized trials, observational studies, animal experiments, *in vitro* research, review articles, commentaries, editorials, case reports, and conference abstracts. For multiple reports of the same trial, we selected the most comprehensive publication with the longest follow-up to prevent data duplication.

### 2.2 Search strategy

We systematically searched PubMed, Embase, Web of Science, and the Cochrane Central Register of Controlled Trials from inception to August 1, 2024. As detailed in SDC1, the search strategy combined medical subject headings (MeSH) and free-text terms related to kidney transplantation, osteoporosis, and bone mineral density. We also manually screened reference lists of included studies and relevant systematic reviews to identify additional eligible trials.

### 2.3 Study selection and data extraction

Two independent reviewers screened titles and abstracts, followed by full-text review of potentially eligible studies. Discrepancies were resolved through consensus or consultation with a third reviewer. Data extraction was performed independently by two reviewers using a standardized, pre-piloted form. Extracted information included study characteristics, participant demographics, intervention details, outcome measures, and data required for risk of bias assessment. For crossover trials, we extracted data from the first period only to avoid potential carryover effects. When both per-protocol and intention-to-treat analyses were reported, we prioritized the latter.

### 2.4 Risk of bias assessment

We assessed risk of bias using the revised Cochrane risk-of-bias tool for randomized trials (RoB 2.0) (18),evaluating five domains: randomization process, deviations from intended interventions, missing outcome data, measurement of the outcome, and selection of the reported results. Two reviewers independently assessed each domain, categorizing studies as low risk, some concerns, or high risk of bias. Disagreements were resolved through discussion or arbitration by a senior author. We generated an overall risk of bias judgment for each study based on the assessments of individual domains. We classified a study as “low risk of bias” if all domains were rated as low risk. If one or more domains were rated as high risk, we categorized the study as “high risk of bias”. Studies with one or more domains rated as “some concerns” and no domains rated as high risk were classified as “unclear risk of bias”.

### 2.5 Statistical analysis

We conducted a frequentist NMA using R version 4.3.2 (R Foundation for Statistical Computing, Vienna, Austria) with the *netmeta* package (version 3.2-0) (17). We calculated mean differences (MD) with 95% confidence intervals (CI) for continuous outcomes and odds ratios (OR) with 95% CI for dichotomous outcomes. We first performed traditional pairwise meta-analyses for all direct comparisons using a random-effects model with the inverse-variance method. We assessed statistical heterogeneity using the I² statistic, interpreting I² values as 0-40% (might not be important), 30-60% (moderate heterogeneity), 50-90% (substantial heterogeneity), and 75-100% (considerable heterogeneity) (18).We then estimated indirect comparison effect sizes using the back-calculation method within the network framework and combined direct and indirect evidence using the to generate network estimates for all possible treatment comparisons using a random-effects model. We assessed local inconsistency using the node-splitting method (19–21),which separates evidence for each comparison into direct and indirect components and calculates their difference with corresponding P-values. We generated network plots to visualize evidence structure, where node size represents total participants for each intervention and edge thickness indicates the number of direct comparisons. We ranked interventions using P-scores, representing the mean extent of certainty that one treatment outperforms another, averaged over all competing treatments. We assessed potential small-study effects and publication bias using Egger’s test and funnel plots (22).We conducted all statistical tests as two-sided and considered P-values < 0.05 as statistically significant.

### 2.6 Assessment of certainty of evidence

We employed the Grading of Recommendations, Assessment, Development, and Evaluation (GRADE) approach to assess the certainty of evidence for each outcome (19–21).This comprehensive assessment encompassed evaluations of both direct comparisons from our pairwise meta-analyses and network estimates from our network meta-analysis (22). For direct comparisons, we assessed risk of bias (23),inconsistency (24),indirectness (25),and publication bias (26). We initiated our assessment with high certainty for randomized controlled trials and subsequently rated down based on concerns in these domains (19). For indirect estimates, we began certainty ratings at the lowest rating of the direct comparisons contributing to the most dominant first-order loop. We further rated down if necessary for intransitivity (27).For the certainty of network estimates, we started with the estimate—direct or indirect—that dominated the network estimate (22).If both direct and indirect estimates contributed importantly to the network estimate, we used the higher of the two as our starting point (22).In cases where we detected incoherence, we rated down the certainty of the network estimates and used the estimate—direct or indirect—with the higher certainty evidence as the best estimate of treatment effect. We evaluated imprecision by examining whether the confidence intervals crossed the line of no effect (28).

We categorized the overall certainty of evidence for each outcome as high, moderate, low, or very low (19). High certainty indicates that we are very confident that the true effect lies close to that of the estimate of the effect. Moderate certainty suggests that we are moderately confident in the effect estimate. Low certainty implies that our confidence in the effect estimate is limited, while very low certainty indicates that we have very little confidence in the effect estimate (19–21).

## 3 Results

### 3.1 Literature screening process and results

**Figure 1:**
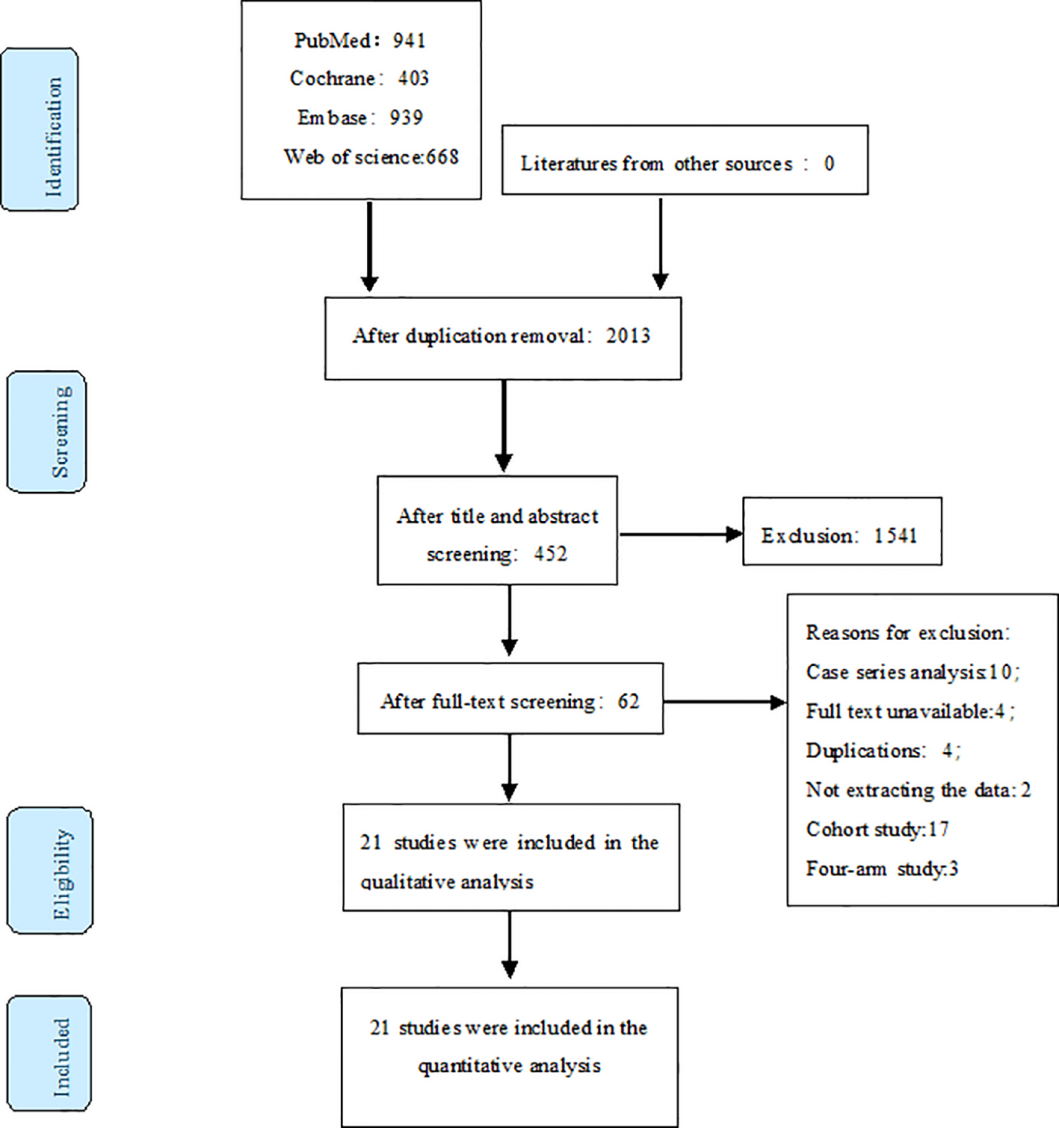
The prisma flow chart of literature screening.

Our initial search yielded 2951 records. After removing duplicates, 2013 articles remained. We screened titles and abstracts, resulting in 452 potentially eligible studies. Full-text review further narrowed this to 62 articles. Finally, we included 21 RCTs in our analysis, as illustrated in **Figure 1**.

### 3.2 Characteristics of included studies

All included studies were RCTs, published between 2000 and April 20, 2021. We excluded studies with incomplete data unsuitable for statistical analysis, as well as duplicate publications, reviews, conference abstracts, and animal experiments. **Table 1** presents the baseline characteristics of the 21 included RCTs (29–40).

**Table 1:** The general information of the included studies.

Studies	Follow-up	Country	Number of patients	Male/female	Age		Intervention	Comparison	Immunosuppression
					Intervention	Comparison			
A. Torres 2004 (30)	12 months	Spain	86	67/19	46.7 ± 12.2	51.1 ± 11.9	Calcitriol	Placebo	Prednisone, Cyclosporine, MMF
Alireza Dabbaghmanesh 2023 (31)	6 months	Iran	33	24/09	51.2 ± 13.53	45.6 ± 15.50	zoledronic acid	Placebo	Prednisone, Cyclosporine, MMF
Amgad E El-Agroudy 2003 (32)	12 months	Egypt	40	40/0	31.4 ± 10.1	31.6 ± 10.7	Alfacalcidol	Placebo	Prednisolone
B Nayak 2007 (33)	6 months	India	50	NA	NA	NA	Alendronate	Blank control	Not mentioned
D Cejka 2008 (60)	6 months	Austria	24	19/5	51 ± 9	54 ± 9	Teriparatide	Placebo	Cyclosporine A, Sirolimus, Tacrolimus, MPA
Igor Denizarde Bacelar Marques 2019 (34)	12 months	Brazil	32	19/13	43 ± 11	39 ± 11	zoledronic acid	Blank control	Glucocorticoids, Tacrolimus, EC-MPS
J-V Torregrosa 2011 (35)	12 months	Spain	39	26/13	53.9 ± 13.79	56.53 ± 15.48	Pamidronate	Placebo	Corticosteroids, MMF, CsA
John R Jeffery 2003 (36)	12 months	Canada	117	71/26	44.8 ± 11.6	45.9 ± 10.8	Alendronate	Calcitriol	Corticosteroids, CsA, AZA or MMF
K T Smerud 2012 (37)	12 months	Norway	129	99/30	50.2 ± 13.5	52.6 ± 14.0	Ibandronate	Placebo	Corticosteroids, MMF, CsA or FK506
M Bonani 2016 (38)	12 months	Switzerland	90	NA	49.0 ± 12.9	50.0 ± 14.0	Denosumab	Blank control	Phosphatase, Corticosteroids, MMF
Makoto Tsujita 2022 (39)	12 months	Japan	211	129/82	52 ± 50.7	52 ± 46.64	Cholecalciferol	Placebo	Prednisolone,Cyclosporine, or Tacrolimus, MMF
Maria Coco 2012 (40)	12 months	USA	42	27/15	42 ± 11	48 ± 14	Risedronate	Placebo	Corticosteroids, MMF, FK506
Martin Haas 2003	6 months	Austria	20	12/8	55 ± 18	49 ± 16	zoledronic acid	Placebo	Corticosteroids, MMF, CsA
Michelle A Josephson 2004	12 months	USA	29	NA	NA	NA	Calcitriol	Placebo	Cyclosporine, Tacrolimus
M Koc 2002	12 months	Turkey	24	17/7	34.4 ± 8.9/40.5 ± 8.1	35.5 ± 8.4	Alendronate/Calcitriol	Blank control	Corticosteroids, AZA, CsA
S Giannini 2001	12 months	Italy	40	27/13	NA	NA	Alendronate	Calcitriol	Cyclosporine, Azathioprine, CsA, MP
S L Fan 2000	12 months	UK	26	26/0	53 ± 37.24	50 ± 40.13	Pamidronate	Placebo	Corticosteroids, CsA with or without AZA
Stanley L-S Fan 2003	12 months	UK	17	NA	46.2 ± 29.92	41.5 ± 26.14	Pamidronate	Placebo	Cyclosporine
Stephen B Walsh 2009	24 months	UK	125	69/24	46.1 ± 12.77	46.1 ± 12.93	Pamidronate	Vitamin D	Corticosteroids, CsA
W H Grotz 1998	12 months	Germany	46	29/17	45 ± 12/14 ± 12	48 ± 12	Sodium clodronate/Calcitonin	Calcium	Corticosteroids, CsA
Wolfgang Grotz 2001	12 months	Germany	80	48/24	NA	NA	Ibandronate	Blank control	Corticosteroids, MMF, CsA

### 3.3 Risk of bias assessment results

**Figure 2:**
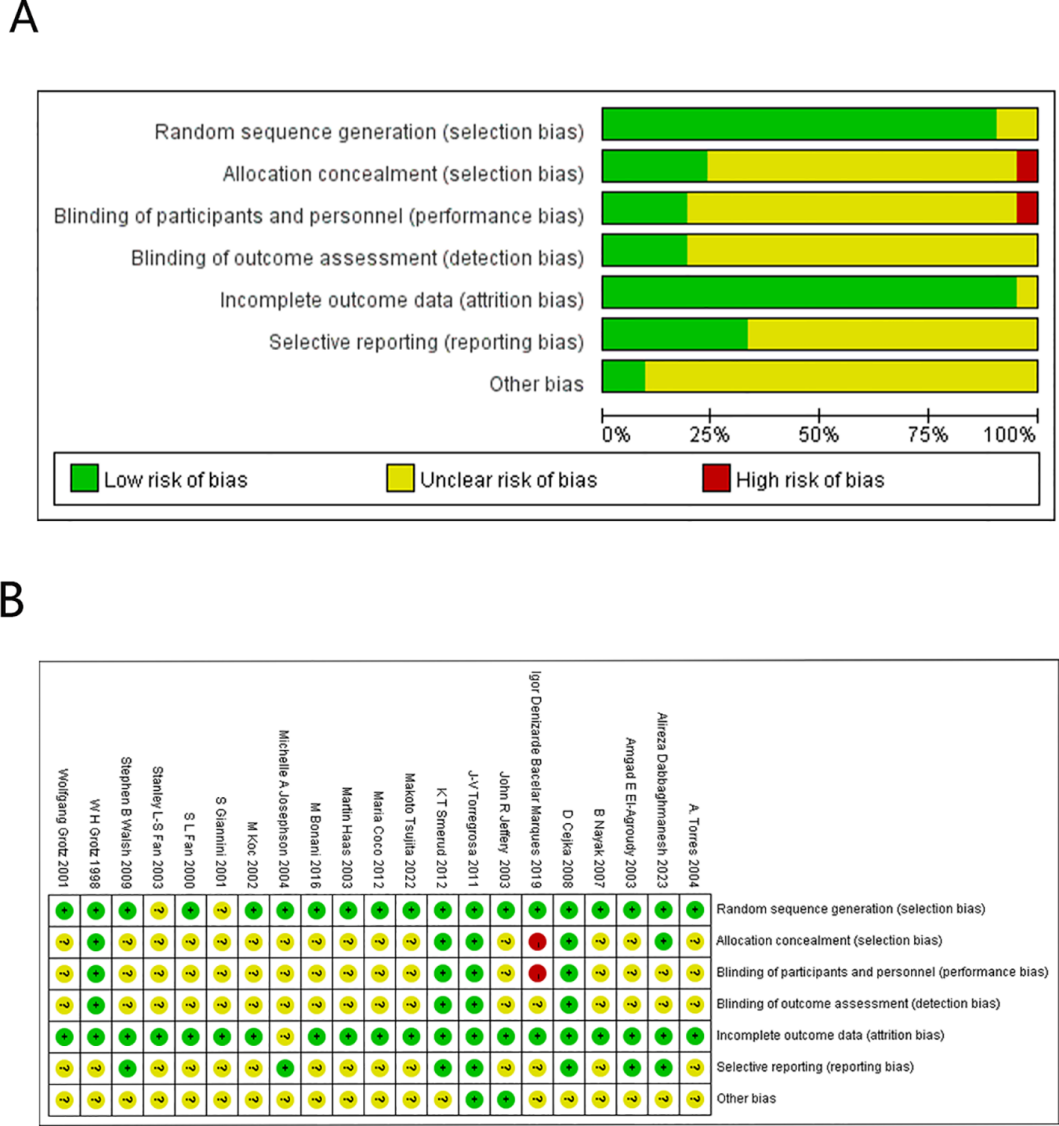
Overall risk of bias. **(A)** Cochrane Bias Risk Percentage Chart. **(B)** Cochrane Bias Risk Summary Chart.

Overall, we classified 2 studies as having a high risk of bias, while 19 studies had low risk of bias for most items. However, 17 studies had some concerns in certain domains. **Figure 2** provides a visual representation of the overall risk of bias.

### 3.4 Network evidence diagram

**Figure 3:**
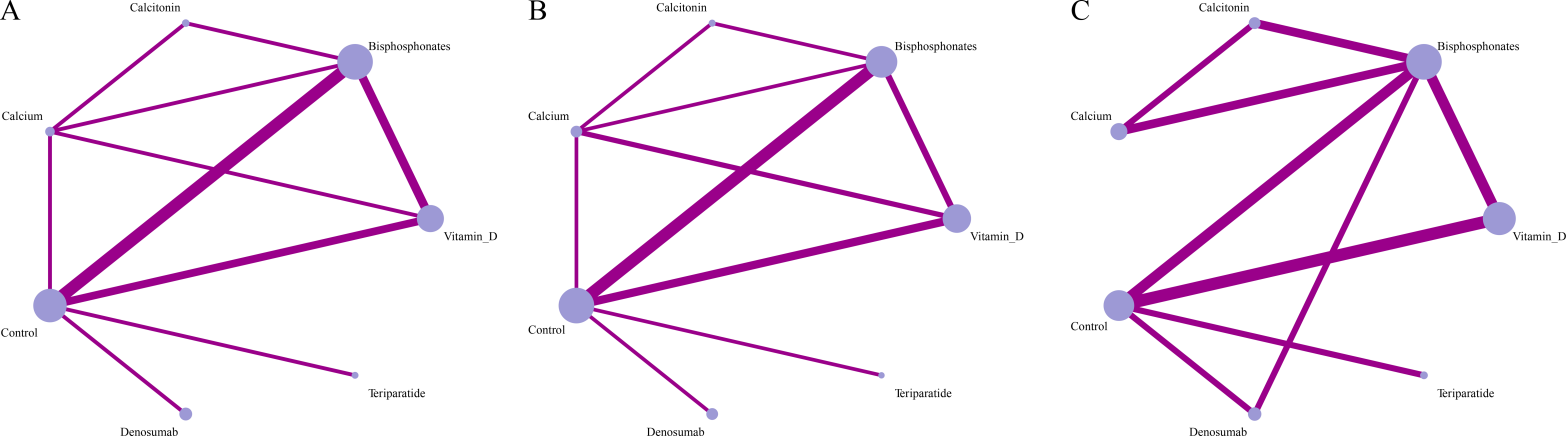
Network meta-analysis results **(A)** g lumbar spine, **(B)** g femoral neck, **(C)** adverse reactions.

The 21 studies reported on three outcomes, resulting in three network evidence diagrams. All outcome indicators showed well-connected network evidence diagrams, as depicted in **Figure 3**. The NMA satisfied the fundamental assumptions of transitivity, consistency, and homogeneity required for valid network synthesis, as detailed in our GRADE assessments (Appendix 7) and inconsistency evaluations (Appendix 5).

### 3.5 Network meta-analysis results

**Table 2** presents the detailed results for all comparisons on three outcomes. Appendix 2 presents the results of pairwise meta-analysis for each comparison. Appendix 3 presents the comprehensive network meta-analysis results for all outcomes. Appendix 4 shows the forest plots of network meta-analysis. Appendix 5 presents the forest plots comparing direct, indirect, and network evidence for each outcome. Appendix 6 presents the funnel plots for assessment of publication bias. Appendix 7 shows the detailed assessments of the certainty of evidence for direct, indirect, and network estimates using the GRADE approach.

**Table 2:** Results of pairwise comparisons.

g/cm² Femoral neck [MD (95%CI)]							
	Bisphosphonates	Calcitonin	Calcium	Denosumab	Teriparatide	Vitamin D	Control
Bisphosphonates	–	0.05 (-0.10, 0.20)	-0.15 (-0.30, 0.00)	–	–	0.03 (-0.04, 0.10)	0.04 (-0.01, 0.09)
Calcitonin	0.12 (-0.02, 0.25)	–	-0.20 (-0.34, -0.06)*	–	–	–	–
Calcium	-0.03 (-0.12, 0.06)	-0.14 (-0.28, -0.01)*	–	–	–	-0.04 (-0.16, 0.09)	-0.01 (-0.13, 0.12)
Denosumab	-1.06 (-3.17, 1.05)	-1.17 (-3.29, 0.94)	-1.03 (-3.14, 1.08)	–	–	–	1.10 (-1.01, 3.21)
Teriparatide	0.01 (-0.28, 0.30)	-0.10 (-0.42, 0.22)	0.04 (-0.26, 0.34)	1.07 (-1.06, 3.20)	–	–	0.03 (-0.26, 0.32)
Vitamin D	-0.00 (-0.06, 0.05)	-0.12 (-0.26, 0.02)	0.03 (-0.07, 0.12)	1.05 (-1.06, 3.17)	-0.02 (-0.31, 0.28)	–	0.07 (0.00, 0.14)*
Control	0.04 (0.00, 0.09)*	-0.07 (-0.21, 0.07)	0.07 (-0.02, 0.16)	1.10 (-1.01, 3.21)	0.03 (-0.26, 0.32)	0.05 (-0.01, 0.10)	–
g/cm² Lumbar spine [MD (95%CI)]							
	Bisphosphonates	Calcitonin	Calcium	Denosumab	Teriparatide	Vitamin D	Control
Bisphosphonates	–	0.01 (-0.33, 0.35)	2.92 (2.49, 3.35)*	–	–	0.07 (-0.12, 0.27)	0.02 (-0.09, 0.12)
Calcitonin	-0.63 (-0.95, -0.31)*	–	2.91 (2.48, 3.34)*	–	–	–	–
Calcium	0.86 (0.62, 1.11)*	1.49 (1.14, 1.85)*	–	–	–	0.23 (-0.10, 0.55)	0.04 (-0.29, 0.38)
Denosumab	-4.98 (-6.84, -3.13)*	-4.35 (-6.24, -2.47)*	-5.85 (-7.72, -3.98)*	–	–	–	5.10 (3.25, 6.95)*
Teriparatide	0.27 (-0.21, 0.74)	0.90 (0.33, 1.46)*	-0.60 (-1.12, -0.07)*	5.25 (3.34, 7.16)*	–	–	-0.15 (-0.61, 0.31)
Vitamin D	0.19 (0.05, 0.33)*	0.82 (0.48, 1.16)*	-0.67 (-0.92, -0.42)*	5.17 (3.32, 7.03)*	-0.08 (-0.56, 0.40)	–	0.02 (-0.14, 0.18)
Control	0.12 (0.02, 0.21)*	0.75 (0.41, 1.08)*	-0.75 (-0.99, -0.50)*	5.10 (3.25, 6.95)*	-0.15 (-0.61, 0.31)	-0.07 (-0.21, 0.06)	–
Adverse Events [OR (95%CI)]							
	Bisphosphonates	Calcitonin	Calcium	Denosumab	Teriparatide	Vitamin D	Control
Bisphosphonates	–	0.52 (0.11, 2.49)	2.44 (0.68, 8.71)	0.90 (0.32, 2.53)	–	0.74 (0.23, 2.41)	0.67 (0.28, 1.59)
Calcitonin	0.71 (0.17, 3.03)	–	2.36 (0.36, 15.45)	–	–	–	–
Calcium	2.52 (0.72, 8.80)	3.53 (0.74, 16.94)	–	–	–	–	–
Denosumab	0.94 (0.35, 2.50)	1.32 (0.23, 7.56)	0.37 (0.08, 1.83)	–	–	–	0.67 (0.20, 2.25)
Teriparatide	0.68 (0.03, 13.47)	0.95 (0.03, 26.34)	0.27 (0.01, 6.87)	0.72 (0.03, 15.70)	–	–	1.00 (0.06, 18.08)
Vitamin D	0.72 (0.30, 1.71)	1.01 (0.19, 5.47)	0.29 (0.06, 1.31)	0.77 (0.23, 2.56)	1.07 (0.05, 21.42)	–	0.95 (0.38, 2.41)
Control	0.68 (0.32, 1.44)	0.95 (0.19, 4.85)	0.27 (0.06, 1.16)	0.72 (0.25, 2.07)	1.00 (0.06, 18.08)	0.94 (0.43, 2.05)	–

In each comparison, the lower left half shows network meta-analysis (NMA) results, and the upper right half shows direct comparison results; “*” indicates P<0.05; “-” indicates data not available or not applicable; MD, Mean Difference; OR, Odds Ratio; CI, Confidence Interval.

### 3.6 Femoral neck bone mineral density

Twenty trials with 1,028 participants reported on femoral neck BMD. Low certainty evidence suggested that bisphosphonates significantly improved femoral neck BMD compared to control (MD = 0.04, 95%CI=0.00, 0.09, p<0.05). Calcitonin was significantly superior to calcium (MD=-0.14, 95%CI=-0.28, -0.01). For other comparisons, Moderate certainty evidence showed no significant differences between bisphosphonates and calcitonin (MD = 0.12, 95%CI=-0.02, 0.25), bisphosphonates and calcium (MD=-0.03, 95%CI=-0.12, 0.06), calcitonin and calcium (MD=-0.14, 95%CI=-0.28, -0.01), calcitonin and teriparatide (MD=-0.10, 95%CI=-0.42, 0.22), and teriparatide and control (MD = 0.03, 95%CI=-0.26, 0.32). Other comparisons showed no statistically significant differences based on very low to low certainty evidence.

P-Score ranking results showed calcitonin (0.89), teriparatide (0.53), bisphosphonates (0.44), vitamin D (0.43), calcium (0.32), and denosumab (0.16), but no statistically significant pairwise differences were found between interventions based on very low to moderate certainty evidence.

### 3.7 Lumbar spine bone mineral density

Twenty-one trials with 1,066 participants reported on lumbar spine BMD. Low certainty evidence showed that denosumab significantly improved lumbar spine BMD compared to control (MD = 5.10, 95%CI=3.25, 6.95, p<0.05). Very low to low certainty evidence indicated that compared to denosumab, other interventions showed significantly lower efficacy: bisphosphonates (MD=-4.98, 95%CI=-6.84, -3.13), calcitonin (MD=-4.35, 95%CI=-6.24, -2.47), and calcium (MD=-5.85, 95%CI=-7.72, -3.98), while denosumab was superior to vitamin D (MD = 5.17, 95%CI=3.32, 7.03) and teriparatide (MD = 5.25, 95%CI=3.34, 7.16). Low certainty evidence also showed that teriparatide was significantly inferior to calcium (MD=-0.60, 95%CI=-1.12, -0.07). For other comparisons, moderate certainty evidence showed no significant differences between bisphosphonates and calcitonin (MD = 0.01, 95%CI=-0.33, 0.35), bisphosphonates and calcium (MD = 2.92, 95%CI=2.49, 3.35), calcitonin and calcium (MD = 2.91, 95%CI=2.48, 3.34), calcitonin and teriparatide (MD = 0.90, 95%CI=0.33, 1.46), calcium and control (MD = 0.04, 95%CI=-0.29, 0.38), and teriparatide and control (MD=-0.15, 95%CI=-0.61, 0.31). Other comparisons showed no statistically significant differences based on very low to low certainty evidence.

P-Score ranking results showed denosumab (1.00), calcitonin (0.83), bisphosphonates (0.64), control (0.44), vitamin D (0.29), teriparatide (0.29), and calcium (0.00). Denosumab demonstrated significantly superior improvement in lumbar spine BMD compared to other interventions, although only one RCT provided direct evidence with low certainty.

### 3.8 Safety

Fourteen studies with 822 participants reported on adverse events. We found no statistically significant differences between interventions, based on low to moderate certainty evidence.

P-Score ranking for safety profile showed control as the safest intervention (P-score=0.69), followed by vitamin D (0.63), calcitonin (0.62), teriparatide (0.58), denosumab (0.47), bisphosphonates (0.41), and calcium (0.09).

## 4 Discussion

Osteoporosis is highly prevalent among KTRs, primarily due to long-term immunosuppressive therapy and post-transplant renal insufficiency. Current guidelines and expert consensus provide crucial references for clinical practice. Bisphosphonates are widely used but require cautious application in KTRs due to potential renal adverse effects. Vitamin D and calcium supplementation are particularly important as fundamental treatments, given the common vitamin D deficiency in KTRs (41). Teriparatide has shown efficacy in KTRs with severe osteoporosis or poor bisphosphonate tolerance. Strontium ranelate may serve as an alternative, though its safety and efficacy in renal impairment require further investigation (42).

Guidelines from the National Osteoporosis Foundation (NOF), Kidney Disease: Improving Global Outcomes (KDIGO), and the European Renal Association - European Dialysis and Transplant Association (ERA-EDTA) recommend regular BMD monitoring for KTRs and timely use of anti-osteoporotic drugs for those at risk of bone loss (43, 44).These guidelines emphasize tailoring treatment regimens based on patients’ renal function, particularly advocating early preventive therapy for patients on long-term glucocorticoid therapy. Chinese expert consensus similarly proposes individualized prevention and treatment of post-transplant bone disease, emphasizing vitamin D and calcium supplementation, as well as the application of bisphosphonates, and recommends regular BMD monitoring when renal function permits (45).

The significant individual variations in osteoporosis risk among KTRs necessitate comprehensive consideration of factors such as renal function and drug tolerability when formulating treatment plans. Although some novel drugs (e.g., deoxycholic acid) have shown promising efficacy in KTRs, their long-term safety and cost-effectiveness require further verification (46, 47).Currently, the application of anti-osteoporotic therapy in KTRs remains in an exploratory stage, with guidelines and expert consensus continuously updating to optimize treatment strategies and further improve patients’ long-term skeletal health.

Our analysis of 21 randomized controlled trials involving 1,066 participants offers important insights into the comparative effectiveness and safety of various interventions for managing post-transplant bone disease, with findings that both support and challenge current clinical practice guidelines. The most striking finding from our analysis is the superior efficacy of denosumab in improving bone mineral density across skeletal sites in KTRs. For lumbar spine BMD, denosumab achieved the highest P-score ranking (1.00) and demonstrated statistically significant superiority over all other interventions, including bisphosphonates, calcitonin, calcium, vitamin D, and control groups, with a substantial mean difference of 5.10% compared to control. While the evidence certainty was low to very low, the consistency of denosumab’s superior performance across outcomes suggests genuine clinical benefit. This finding is particularly relevant given denosumab’s unique mechanism of action as a RANKL inhibitor that effectively suppresses osteoclast activity without requiring renal elimination—a crucial advantage in KTRs where residual renal impairment is common and may compromise the efficacy and safety of renally-eliminated medications like bisphosphonates. While a previous network meta-analysis compared only six bisphosphonates’ effects on BMD in KTRs (48), our study is the first to incorporate vitamin D, bisphosphonates, denosumab, teriparatide, calcitonin, and calcium into a network meta-analysis, addressing the lack of direct comparisons between different treatment modalities (49).

In the ranking of efficacy for improving femoral neck BMD, denosumab and calcium topped the list, followed by vitamin D. Denosumab, a RANKL inhibitor, effectively suppresses osteoclast activity and increases bone density without being affected by renal function, thus demonstrating superior efficacy in the femoral neck region of KTRs (48, 50, 51).Calcium supplementation, primarily used to ameliorate hypocalcemia and maintain bone health, has limited efficacy when used alone and typically requires combination with other drugs (52). Vitamin D aids in regulating calcium and phosphorus metabolism and promoting bone mineralization, usually serving as a fundamental supportive therapy (53, 54). For lumbar spine BMD improvement, denosumab and calcitonin ranked highest, followed by bisphosphonates. Denosumab significantly increases lumbar spine bone density by inhibiting the RANKL/RANK signaling pathway, representing an efficient and safe long-term treatment option unaffected by renal function (55). Calcitonin, used for short-term treatment, has moderate effects and may lead to drug resistance with prolonged use, particularly showing less significant effects on the lumbar spine compared to denosumab (56). Bisphosphonates demonstrate significant improvement in lumbar spine BMD but require cautious use in patients with impaired renal function due to their renal excretion, to avoid nephrotoxicity (57, 58). However, the P-score rankings presented in this analysis should be considered as supplementary reference information rather than definitive therapeutic hierarchies. While these probabilistic rankings provide insight into the relative positioning of treatments within the evidence network, clinical decision-making should prioritize statistically significant direct and indirect comparisons over ranking positions, particularly given the varying certainty of evidence across different treatment comparisons.

From clinical nephrology and transplantation perspectives, denosumab offers unique pharmacokinetic advantages as a RANKL inhibitor that undergoes hepatic metabolism rather than renal clearance, allowing for stable drug concentrations in KTRs with impaired graft function without requiring dose adjustments based on renal function fluctuations (37). The interaction between anti-osteoporotic therapies and immunosuppressive regimens remains complex, as calcineurin inhibitors such as tacrolimus and cyclosporine can induce hypercalciuria and hypomagnesemia, potentially affecting bone metabolism and the efficacy of bone-targeting treatments (59, 60). Denosumab therapy in KTRs necessitates vigilant monitoring for infection risks, particularly urinary tract infections, since immunosuppressed patients may experience increased susceptibility to infections due to the dual impact of RANKL inhibition on immune cell development and ongoing immunosuppressive therapy. The choice of immunosuppressive regimen significantly influences fracture risk, with steroid-sparing protocols showing superior bone outcomes compared to traditional triple therapy regimens, highlighting the importance of integrated nephrology and transplantation approaches to optimize both graft survival and bone health (61).

While our analysis found no statistically significant differences between interventions for safety outcomes, several important considerations warrant discussion regarding the clinical interpretation of these findings in KTRs. The wide confidence intervals observed for adverse event comparisons reflect the sparse nature of safety data, with only 14 studies involving 822 participants reporting adverse events. This limited safety evidence is particularly concerning for KTRs, who represent a uniquely vulnerable population already at heightened risk for complications due to chronic immunosuppression, residual renal impairment, and altered mineral metabolism. The immunocompromised state of KTRs may predispose them to increased infection risks with certain treatments like denosumab, which can further suppress immune function through its effects on the RANKL/RANK/OPG pathway. Additionally, KTRs’ altered mineral metabolism increases their susceptibility to hypocalcemia, particularly with denosumab therapy, potentially leading to serious clinical consequences. The relatively short follow-up periods in most studies (typically 12–24 months) may be insufficient to capture long-term safety signals that are particularly relevant for KTRs requiring lifelong anti-osteoporotic therapy, such as osteonecrosis of the jaw, atypical fractures, or cardiovascular events associated with calcium supplementation (62). Furthermore, potential interactions between anti-osteoporotic medications and immunosuppressive regimens remain poorly characterized in the available literature. The heterogeneity in immunosuppressive protocols across studies limits our ability to make specific safety recommendations for different immunosuppressive combinations, highlighting the need for individualized risk-benefit assessments in clinical practice.

These findings have important implications for clinical practice and guideline development. Current guidelines emphasizing bisphosphonates as first-line therapy may need revision to incorporate the emerging evidence supporting denosumab’s superior efficacy, particularly for KTRs with compromised renal function. A risk-stratified approach appears most appropriate: high-risk patients with severe osteoporosis or previous fractures might benefit from denosumab as first-line therapy with careful monitoring, while moderate-risk patients with adequate renal function could continue with bisphosphonates as first-line treatment, with denosumab reserved for those with renal impairment or bisphosphonate intolerance. All patients should receive adequate calcium and vitamin D supplementation as foundational therapy, with treatment decisions individualized based on renal function, infection risk, and overall health status. Several important limitations must be acknowledged when interpreting these results. The certainty of evidence ranged from very low to moderate for most comparisons, limiting the strength of our conclusions and highlighting the need for higher-quality studies. Significant heterogeneity existed across studies in terms of patient populations, transplant vintage, immunosuppressive regimens, and follow-up duration, which may have influenced our findings. The limited number of studies for some interventions, particularly denosumab (only one RCT), restricts the robustness of our conclusions and emphasizes the need for additional high-quality trials. Furthermore, our analysis focused on BMD changes rather than fracture outcomes, which are more clinically relevant but were insufficiently reported in the included studies to allow meaningful analysis. Future research should prioritize large-scale, long-term randomized controlled trials directly comparing denosumab with bisphosphonates in KTRs, with fracture reduction as the primary endpoint. Studies examining optimal timing of anti-osteoporotic therapy initiation post-transplant, investigation of combination therapies and sequential treatment strategies, real-world effectiveness studies examining long-term safety and adherence patterns, and cost-effectiveness analyses to inform healthcare policy decisions are all needed to advance the field and improve patient outcomes.

## 5 Conclusions

Our network meta-analysis provides the most comprehensive evidence synthesis to date regarding anti-osteoporotic therapies in KTRs, with denosumab appearing to be the most effective intervention for improving BMD while calcium supplementation offers the best safety profile. However, the moderate to low certainty of evidence and the complexity of post-transplant bone disease necessitate individualized treatment approaches that carefully consider patient-specific factors. These findings suggest that current clinical guidelines may benefit from revision to incorporate emerging evidence supporting denosumab’s efficacy, though treatment decisions should continue to integrate efficacy findings with careful consideration of individual patient characteristics, safety profiles, and clinical preferences until larger, longer-term studies confirm these findings and establish fracture reduction benefits.

## Data Availability

The original contributions presented in the study are included in the article/**Supplementary Material**. Further inquiries can be directed to the corresponding authors.
