# Supplementary material for: Comparative efficacy and safety of anti-osteoporotic therapies for kidney transplant recipients: a systematic review and network meta-analysis
Source: Front Endocrinol (Lausanne). 2025 Nov 3;16:1689233. doi: 10.3389/fendo.2025.1689233 (PMC12620273; doi:10.3389/fendo.2025.1689233)
Supplement: Supplementary file 1 [file DataSheet1.pdf]

SUPPLEMENTARY APPENDIX

Comparative Efficacy and Safety of Anti-Osteoporotic Therapies for Kidney Transplant Recipients: A Systematic Review and Network Meta-Analysis

Table of contents

1 FULL SEARCH STRATEGY 3

1.1 PubMed *3*

1.2 Embase *5*

1.3 Web of Science *6*

1.4 Cochrane Central Register of Controlled Trials *8*

2 RESULTS OF PAIRWISE META-ANALYSIS 9

2.1 Femoral neck bone mineral density *9*

2.2 Lumbar spine bone mineral density *10*

2.3 Adverse events *11*

3 RESULTS OF NETWORK META-ANALYSIS 12

3.1 Femoral neck bone mineral density *12*

3.2 Lumbar spine bone mineral density *13*

3.3 Adverse events *14*

4 FOREST PLOTS OF NETWORK META-ANALYSIS 15

4.1 Femoral neck bone mineral density *15*

4.2 Lumbar spine bone mineral density *16*

4.3 Adverse events *17*

5 FOREST PLOTS OF DIRECT, INDIRECT, AND NETWORK EVIDENCE 18

5.1 Femoral neck bone mineral density *18*

5.2 Lumbar spine bone mineral density *19*

5.3 Adverse events *20*

6 FUNNEL PLOTS 21

6.1 Femoral neck bone mineral density *21*

6.2 Lumbar spine bone mineral density *22*

6.3 Adverse events *23*

7 RESULTS OF ASSESSMENTS OF THE CERTAINTY OF EVIDENCE FOR DIRECT, INDIRECT AND NETWORK ESTIMATES 24

7.1 Femoral neck bone mineral density *24*

7.2 Lumbar spine bone mineral density *26*

7.3 Adverse events *28*

1 Full search strategy

1.1 PubMed

#	Search Details	Results
**1**	"kidney transplantation"[MeSH Terms]	107,127
**2**	"kidney allograft transplantation"[Title/Abstract] OR "kidney allotransplantation"[Title/Abstract] OR "Kidney Grafting"[Title/Abstract] OR "kidney homotransplantation"[Title/Abstract] OR "kidney retransplantation"[Title/Abstract] OR "kidney transplantation"[Title/Abstract] OR "Kidney Transplantations"[Title/Abstract] OR "renal homotransplantation"[Title/Abstract] OR "renal transplantation"[Title/Abstract] OR "Renal Transplantations"[Title/Abstract]	60,363
**3**	"bone density"[MeSH Terms]	62,312
**4**	"Bone Densities"[Title/Abstract] OR "bone density"[Title/Abstract] OR "Bone Mineral Content"[Title/Abstract] OR "Bone Mineral Contents"[Title/Abstract] OR "Bone Mineral Densities"[Title/Abstract] OR "bone mineral density"[Title/Abstract] OR "osseous density"[Title/Abstract] OR "T score"[Title/Abstract] OR "Osteocalcin"[Title/Abstract] OR "P1NP"[Title/Abstract] OR "CTX"[Title/Abstract] OR "NTX"[Title/Abstract] OR "Alkaline Phosphatase"[Title/Abstract]	171,538
**5**	("kidney transplantation"[MeSH Terms] OR ("kidney allograft transplantation"[Title/Abstract] OR "kidney allotransplantation"[Title/Abstract] OR "Kidney	941

	Grafting"[Title/Abstract] OR "kidney homotransplantation"[Title/Abstract] OR "kidney retransplantation"[Title/Abstract] OR "kidney transplantation"[Title/Abstract] OR "Kidney Transplantations"[Title/Abstract] OR "renal homotransplantation"[Title/Abstract] OR "renal transplantation"[Title/Abstract] OR "Renal Transplantations"[Title/Abstract])) AND ("bone density"[MeSH Terms] OR ("Bone Densities"[Title/Abstract] OR "bone density"[Title/Abstract] OR "Bone Mineral Content"[Title/Abstract] OR "Bone Mineral Contents"[Title/Abstract] OR "Bone Mineral Densities"[Title/Abstract] OR "bone mineral density"[Title/Abstract] OR "osseous density"[Title/Abstract] OR "T score"[Title/Abstract] OR "Osteocalcin"[Title/Abstract] OR "P1NP"[Title/Abstract] OR "CTX"[Title/Abstract] OR "NTX"[Title/Abstract] OR "Alkaline Phosphatase"[Title/Abstract]))	

1.2 Embase

#	Search Details	Results
**1**	'kidney transplantation'/exp	195187
**2**	'kidney allograft transplantation':ti,ab,kw OR 'kidney allotransplantation':ti,ab,kw OR 'kidney cadaver transplantation':ti,ab,kw OR 'kidney grafting':ti,ab,kw OR 'kidney homotransplantation':ti,ab,kw OR 'kidney retransplantation':ti,ab,kw OR 'kidney transplantation':ti,ab,kw OR 'kidney transplantations':ti,ab,kw OR 'renal homotransplantation':ti,ab,kw OR 'renal transplantation':ti,ab,kw OR 'renal transplantations':ti,ab,kw OR 'second set kidney transplantation':ti,ab,kw	98077
**3**	'bone density'/exp	119043
**4**	'bone densities':ti,ab,kw OR 'bone density':ti,ab,kw OR 'bone mineral content':ti,ab,kw OR 'bone mineral contents':ti,ab,kw OR 'bone mineral densities':ti,ab,kw OR 'bone mineral density':ti,ab,kw OR 'osseous density':ti,ab,kw OR 't score':ti,ab,kw OR 'osteocalcin':ti,ab,kw OR 'p1np':ti,ab,kw OR 'ctx':ti,ab,kw OR 'ntx':ti,ab,kw OR 'alkaline phosphatase':ti,ab,kw	238133
**5**	(#1 OR #2) AND (#3 OR #4)	1947
**6**	#5 AND 'Article'/it	939

1.3 Web of Science

#	Search Details	Results
**1**	(TI=((“kidney allograft transplantation”) OR (“kidney allotransplantation”) OR (“kidney cadaver transplantation”) OR (“Kidney Grafting”) OR (“kidney homotransplantation”) OR (“kidney retransplantation”) OR (“kidney transplantation”) OR (“Kidney Transplantations”) OR (“renal homotransplantation”) OR (“renal transplantation”) OR (“Renal Transplantations”) OR (“second set kidney transplantation”)) OR AK=((“kidney allograft transplantation”) OR (“kidney allotransplantation”) OR (“kidney cadaver transplantation”) OR (“Kidney Grafting”) OR (“kidney homotransplantation”) OR (“kidney retransplantation”) OR (“kidney transplantation”) OR (“Kidney Transplantations”) OR (“renal homotransplantation”) OR (“renal transplantation”) OR (“Renal Transplantations”) OR (“second set kidney transplantation”)) OR AB=((“kidney allograft transplantation”) OR (“kidney allotransplantation”) OR (“kidney cadaver transplantation”) OR (“Kidney Grafting”) OR (“kidney homotransplantation”) OR (“kidney retransplantation”) OR (“kidney transplantation”) OR (“Kidney Transplantations”) OR (“renal homotransplantation”) OR (“renal transplantation”) OR (“Renal Transplantations”) OR (“second set kidney transplantation”))) AND (TI=((“Bone Densities”) OR (“bone density”) OR (“Bone Mineral Content”) OR (“Bone Mineral Contents”) OR (“Bone Mineral Densities”) OR (“bone mineral density”) OR (“osseous density”) OR (“T score”) OR (“Osteocalcin”) OR (“P1NP”) OR (“CTX”) OR (“NTX”) OR (“Alkaline Phosphatase”)) OR AK=((“Bone Densities”) OR (“bone density”) OR (“Bone Mineral Content”) OR (“Bone	668

	Mineral Contents") OR ("Bone Mineral Densities") OR ("bone mineral density") OR ("osseous density") OR ("T score") OR ("Osteocalcin") OR ("P1NP") OR ("CTX") OR ("NTX") OR ("Alkaline Phosphatase")) OR AB=(("Bone Densities") OR ("bone density") OR ("Bone Mineral Content") OR ("Bone Mineral Contents") OR ("Bone Mineral Densities") OR ("bone mineral density") OR ("osseous density") OR ("T score") OR ("Osteocalcin") OR ("P1NP") OR ("CTX") OR ("NTX") OR ("Alkaline Phosphatase")))	

1.4 Cochrane Central Register of Controlled Trials

#	Search Details	Results
**1**	(‘kidney allograft transplantation’ OR ‘kidney allotransplantation’ OR ‘kidney cadaver transplantation’ OR ‘Kidney Grafting’ OR ‘kidney homotransplantation’ OR ‘kidney retransplantation’ OR ‘kidney transplantation’ OR ‘Kidney Transplantations’ OR ‘renal homotransplantation’ OR ‘renal transplantation’ OR ‘Renal Transplantations’ OR ‘second set kidney transplantation’):ti;ab;kw	12466
**2**	MeSH descriptor: [Kidney Transplantation] explode all trees	4702
**3**	(‘Bone Densities’ OR ‘bone density’ OR ‘Bone Mineral Content’ OR ‘Bone Mineral Contents’ OR ‘Bone Mineral Densities’ OR ‘bone mineral density’ OR ‘osseous density’ OR ‘T score’ OR ‘Osteocalcin’ OR ‘P1NP’ OR ‘CTX’ OR ‘NTX’ OR ‘Alkaline Phosphatase’):ti;ab;kw	43640
**4**	MeSH descriptor: [Bone Density] explode all trees	6022
**5**	(#1 OR #2) AND (#3 OR #4)	406

2 Results of pairwise meta-analysis

2.1 Femoral neck bone mineral

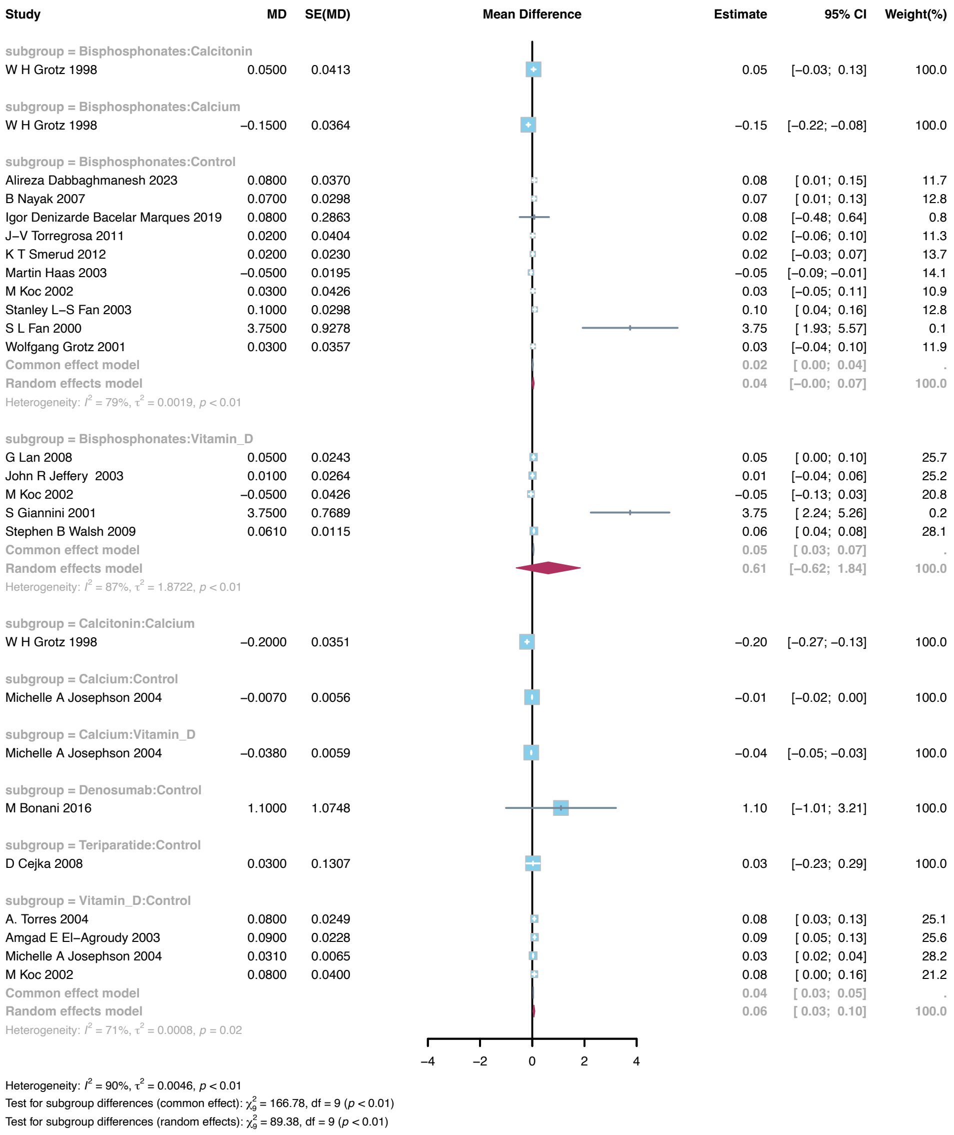

Note: The terms "Common effect model" and "Random effects model" are used as per the default graphical output of the statistical software. "Common effect model" is equivalent to the "fixed-effect model" used in the main text.

2.2 Lumbar spine bone mineral density

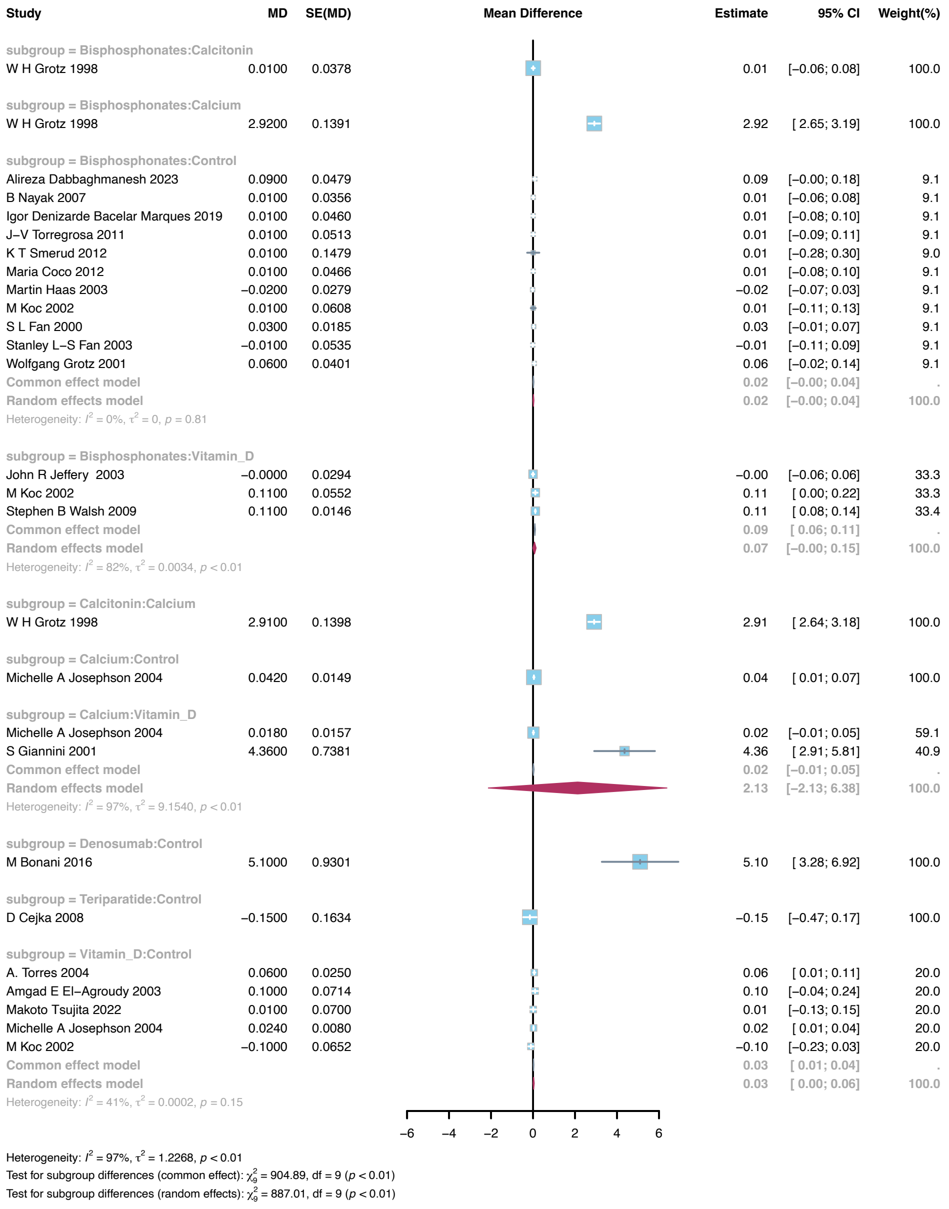

Note: The terms "Common effect model" and "Random effects model" are used as per the default graphical output of the statistical software. "Common effect model" is equivalent to the "fixed-effect model" used in the main text.

2.3 Adverse events

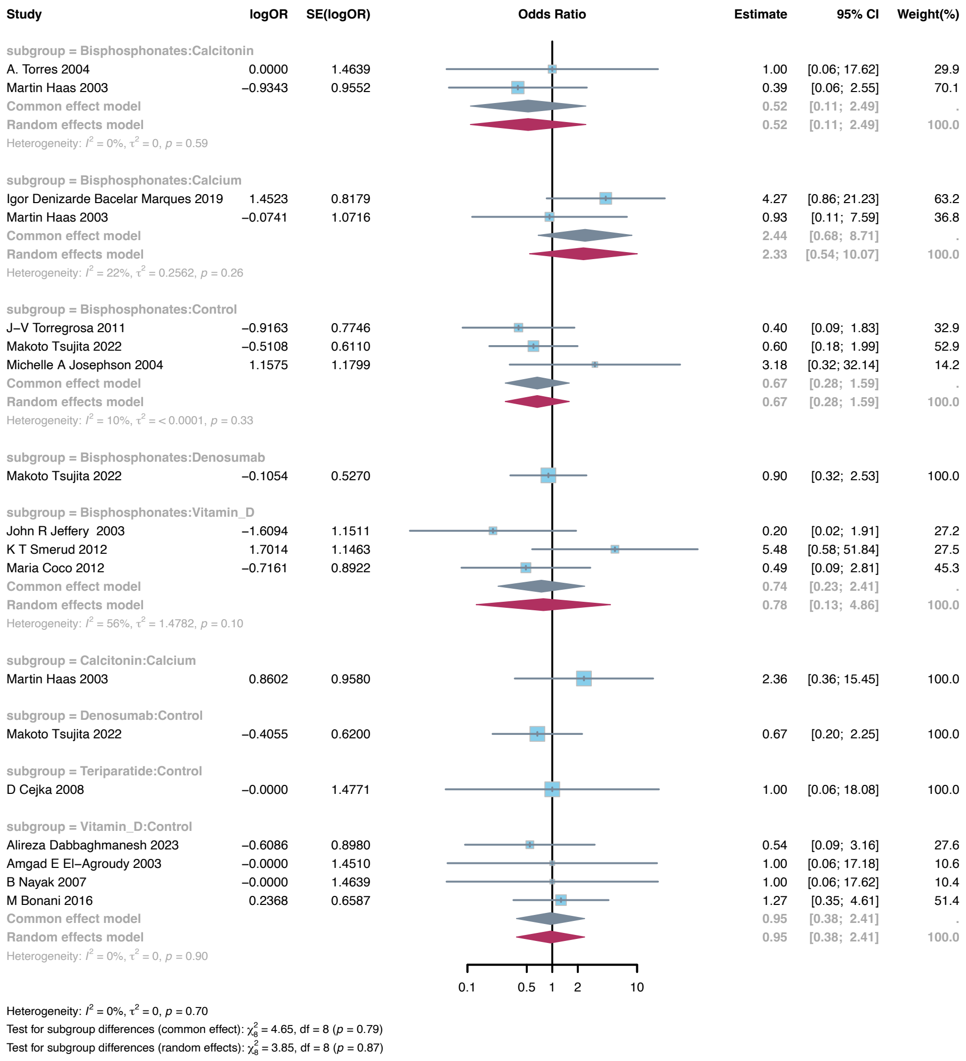

Note: The terms "Common effect model" and "Random effects model" are used as per the default graphical output of the statistical software. "Common effect model" is equivalent to the "fixed-effect model" used in the main text.

3 Results of network meta-analysis

3.1 Femoral neck bone mineral density

Bisphosphonates	0.05 (-0.10 - 0.20)	-0.15 (-0.30 - 0.00)	.	.	0.03 (-0.04 - 0.10)	0.04 (-0.01 - 0.09)
0.12 (-0.02 - 0.25)	Calcitonin	-0.20 (-0.34 - -0.06)	.	.	.	.
-0.03 (-0.12 - 0.06)	**-0.14 (-0.28 - -0.01)**	Calcium	.	.	-0.04 (-0.16 - 0.09)	-0.01 (-0.13 - 0.12)
-1.06 (-3.17 - 1.05)	-1.17 (-3.29 - 0.94)	-1.03 (-3.14 - 1.08)	Denosumab	.	.	1.10 (-1.01 - 3.21)
0.01 (-0.28 - 0.30)	-0.10 (-0.42 - 0.22)	0.04 (-0.26 - 0.34)	1.07 (-1.06 - 3.20)	Teriparatide	.	0.03 (-0.26 - 0.32)
-0.00 (-0.06 - 0.05)	-0.12 (-0.26 - 0.02)	0.03 (-0.07 - 0.12)	1.05 (-1.06 - 3.17)	-0.02 (-0.31 - 0.28)	Vitamin_D	0.07 (0.00 - 0.14)
**0.04 (0.00 - 0.09)**	-0.07 (-0.21 - 0.07)	0.07 (-0.02 - 0.16)	1.10 (-1.01 - 3.21)	0.03 (-0.26 - 0.32)	0.05 (-0.01 - 0.10)	Control

3.2 Lumbar spine bone mineral density

Bisphosphonates	0.01 (-0.33 - 0.35)	2.92 (2.49 - 3.35)	.	.	0.07 (-0.12 - 0.27)	0.02 (-0.09 - 0.12)
**-0.63 (-0.95 - -0.31)**	Calcitonin	2.91 (2.48 - 3.34)	.	.	.	.
0.86 (0.62 - 1.11)	**1.49 (1.14 - 1.85)**	Calcium	.	.	0.23 (-0.10 - 0.55)	0.04 (-0.29 - 0.38)
**-4.98 (-6.84 - -3.13)**	**-4.35 (-6.24 - -2.47)**	**-5.85 (-7.72 - -3.98)**	Denosumab	.	.	5.10 (3.25 - 6.95)
0.27 (-0.21 - 0.74)	**0.90 (0.33 - 1.46)**	**-0.60 (-1.12 - -0.07)**	**5.25 (3.34 - 7.16)**	Teriparatide	.	-0.15 (-0.61 - 0.31)
**0.19 (0.05 - 0.33)**	**0.82 (0.48 - 1.16)**	**-0.67 (-0.92 - -0.42)**	**5.17 (3.32 - 7.03)**	-0.08 (-0.56 - 0.40)	Vitamin_D	0.02 (-0.14 - 0.18)
**0.12 (0.02 - 0.21)**	**0.75 (0.41 - 1.08)**	**-0.75 (-0.99 - -0.50)**	**5.10 (3.25 - 6.95)**	-0.15 (-0.61 - 0.31)	-0.07 (-0.21 - 0.06)	Control

3.3 Adverse events

Bisphosphonates	0.52 (0.11 - 2.49)	2.44 (0.68 - 8.71)	0.90 (0.32 - 2.53)	.	0.74 (0.23 - 2.41)	0.67 (0.28 - 1.59)
0.71 (0.17 - 3.03)	Calcitonin	2.36 (0.36 - 15.45)	.	.	.	.
2.52 (0.72 - 8.80)	3.53 (0.74 - 16.94)	Calcium	.	.	.	.
0.94 (0.35 - 2.50)	1.32 (0.23 - 7.56)	0.37 (0.08 - 1.83)	Denosumab	.	.	0.67 (0.20 - 2.25)
0.68 (0.03 - 13.47)	0.95 (0.03 - 26.34)	0.27 (0.01 - 6.87)	0.72 (0.03 - 15.70)	Teriparatide	.	1.00 (0.06 - 18.08)
0.72 (0.30 - 1.71)	1.01 (0.19 - 5.47)	0.29 (0.06 - 1.31)	0.77 (0.23 - 2.56)	1.07 (0.05 - 21.42)	Vitamin_D	0.95 (0.38 - 2.41)
0.68 (0.32 - 1.44)	0.95 (0.19 - 4.85)	0.27 (0.06 - 1.16)	0.72 (0.25 - 2.07)	1.00 (0.06 - 18.08)	0.94 (0.43 - 2.05)	Control

4 Forest plots of network meta-analysis

4.1 Femoral neck bone mineral density

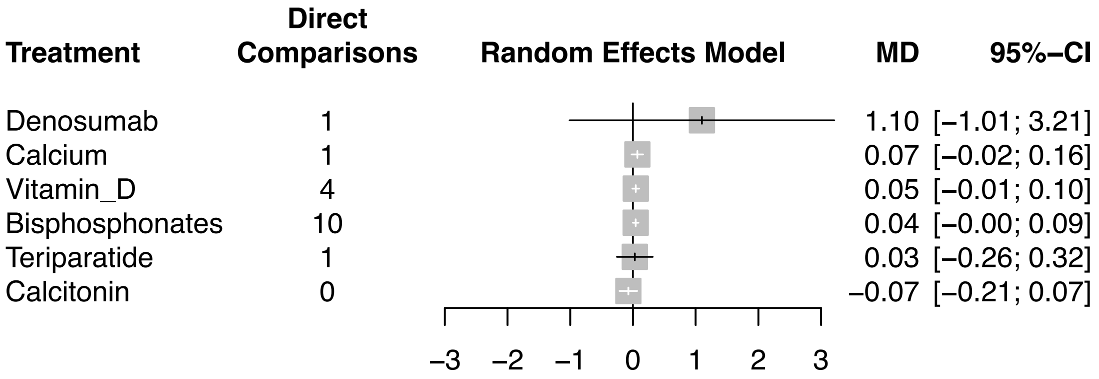

P-scores:
Denosumab: 0.8415;
Calcium: 0.6850;
Vitamin_D: 0.5701;
Bisphosphonates: 0.5644;
Teriparatide: 0.4664;
Control: 0.2606;
Calcitonin: 0.1121

4.2 Lumbar spine bone mineral density

Treatment	Direct Comparisons	MD	95%–CI
Denosumab	1	5.10	[3.25; 6.95]
Calcitonin	0	0.75	[0.41; 1.08]
Bisphosphonates	11	0.12	[0.02; 0.21]
Vitamin_D	5	−0.07	[−0.21; 0.06]
Teriparatide	1	−0.15	[−0.61; 0.31]
Calcium	1	−0.75	[−0.99; −0.50]

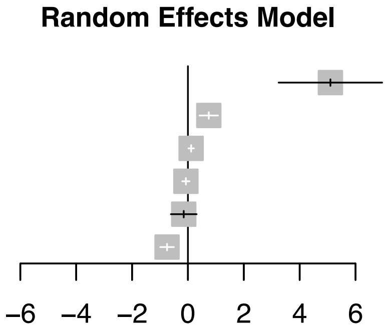

P-scores:
Denosumab: 1.0000;
Calcitonin: 0.8332;
Bisphosphonates: 0.6417;
Control: 0.4351;
Vitamin_D: 0.2941;
Teriparatide: 0.2938;
Calcium: 0.0021

4.3 Adverse events

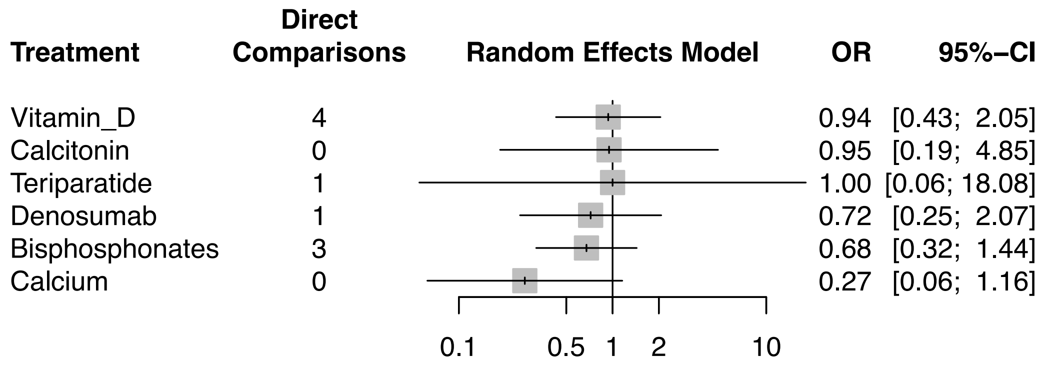

P-scores:

Control: 0.6876;

Vitamin_D: 0.6324;

Calcitonin: 0.6182;

Teriparatide: 0.5833;

Denosumab: 0.4732;

Bisphosphonates: 0.4138;

Calcium: 0.0915

5 Forest plots of direct, indirect, and network evidence

5.1 Femoral neck bone mineral density

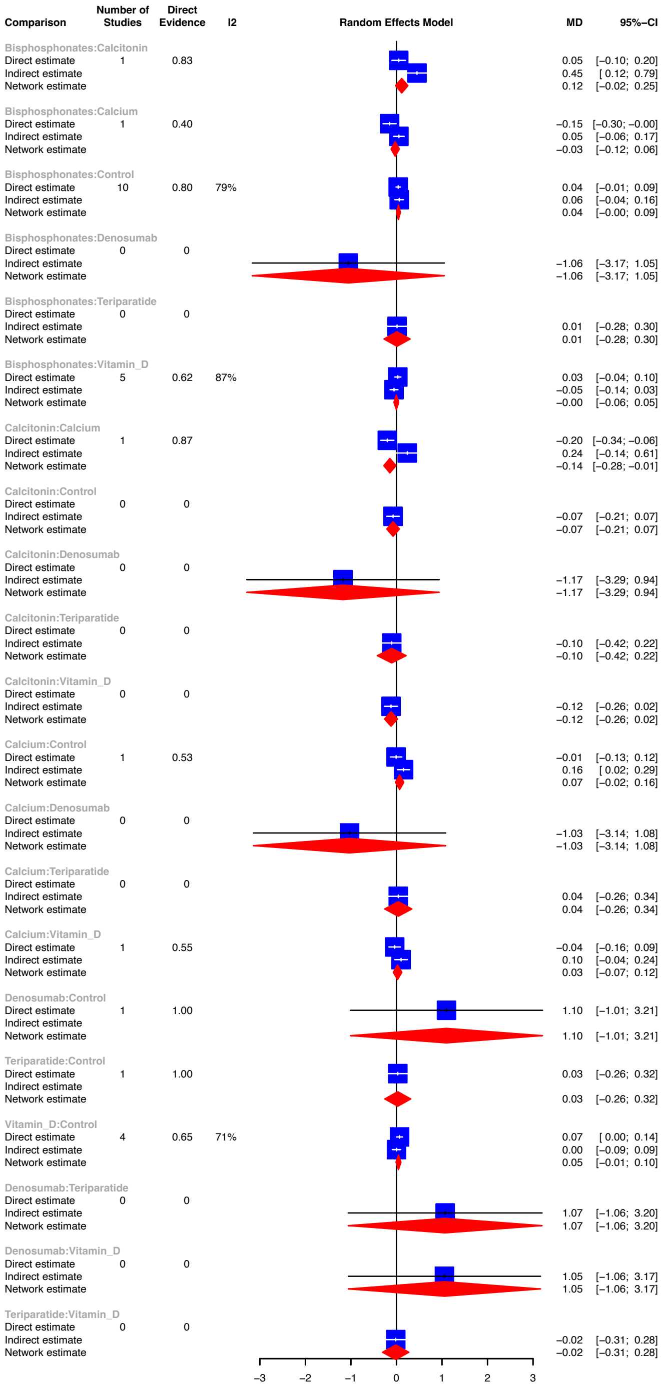

5.2 Lumbar spine bone mineral density

Comparison	Number of Studies	Direct Evidence	I2	MD	95%–CI
**Bisphosphonates:Calcitonin**					
Direct estimate	1	0.90		0.01	[–0.33; 0.35]
Indirect estimate				–6.18	[–7.18; –5.18]
Network estimate				–0.63	[–0.95; –0.31]
**Bisphosphonates:Calcium**					
Direct estimate	1	0.33		2.92	[2.49; 3.35]
Indirect estimate				–0.15	[–0.46; 0.15]
Network estimate				0.86	[0.62; 1.11]
**Bisphosphonates:Control**					
Direct estimate	11	0.86	0%	0.02	[–0.09; 0.12]
Indirect estimate				0.72	[0.45; 0.98]
Network estimate				0.12	[0.02; 0.21]
**Bisphosphonates:Denosumab**					
Direct estimate	0	0			
Indirect estimate				–4.98	[–6.84; –3.13]
Network estimate				–4.98	[–6.84; –3.13]
**Bisphosphonates:Teriparatide**					
Direct estimate	0	0			
Indirect estimate				0.27	[–0.21; 0.74]
Network estimate				0.27	[–0.21; 0.74]
**Bisphosphonates:Vitamin_D**					
Direct estimate	3	0.50	82%	0.07	[–0.12; 0.27]
Indirect estimate				0.31	[0.11; 0.50]
Network estimate				0.19	[0.05; 0.33]
**Calcitonin:Calcium**					
Direct estimate	1	0.68		2.91	[2.48; 3.34]
Indirect estimate				–1.57	[–2.21; –0.94]
Network estimate				1.49	[1.14; 1.85]
**Calcitonin:Control**					
Direct estimate	0	0			
Indirect estimate				0.75	[0.41; 1.08]
Network estimate				0.75	[0.41; 1.08]
**Calcitonin:Denosumab**					
Direct estimate	0	0			
Indirect estimate				–4.35	[–6.24; –2.47]
Network estimate				–4.35	[–6.24; –2.47]
**Calcitonin:Teriparatide**					
Direct estimate	0	0			
Indirect estimate				0.90	[0.33; 1.46]
Network estimate				0.90	[0.33; 1.46]
**Calcitonin:Vitamin_D**					
Direct estimate	0	0			
Indirect estimate				0.82	[0.48; 1.16]
Network estimate				0.82	[0.48; 1.16]
**Calcium:Control**					
Direct estimate	1	0.54		0.04	[–0.29; 0.38]
Indirect estimate				–1.67	[–2.04; –1.31]
Network estimate				–0.75	[–0.99; –0.50]
**Calcium:Denosumab**					
Direct estimate	0	0			
Indirect estimate				–5.85	[–7.72; –3.98]
Network estimate				–5.85	[–7.72; –3.98]
**Calcium:Teriparatide**					
Direct estimate	0	0			
Indirect estimate				–0.60	[–1.12; –0.07]
Network estimate				–0.60	[–1.12; –0.07]
**Calcium:Vitamin_D**					
Direct estimate	2	0.59	97%	0.23	[–0.10; 0.55]
Indirect estimate				–1.97	[–2.36; –1.58]
Network estimate				–0.67	[–0.92; –0.42]
**Denosumab:Control**					
Direct estimate	1	1.00		5.10	[3.25; 6.95]
Indirect estimate					
Network estimate				5.10	[3.25; 6.95]
**Teriparatide:Control**					
Direct estimate	1	1.00		–0.15	[–0.61; 0.31]
Indirect estimate					
Network estimate				–0.15	[–0.61; 0.31]
**Vitamin_D:Control**					
Direct estimate	5	0.72	41%	0.02	[–0.14; 0.18]
Indirect estimate				–0.31	[–0.56; –0.06]
Network estimate				–0.07	[–0.21; 0.06]
**Denosumab:Teriparatide**					
Direct estimate	0	0			
Indirect estimate				5.25	[3.34; 7.16]
Network estimate				5.25	[3.34; 7.16]
**Denosumab:Vitamin_D**					
Direct estimate	0	0			
Indirect estimate				5.17	[3.32; 7.03]
Network estimate				5.17	[3.32; 7.03]
**Teriparatide:Vitamin_D**					
Direct estimate	0	0			
Indirect estimate				–0.08	[–0.56; 0.40]
Network estimate				–0.08	[–0.56; 0.40]

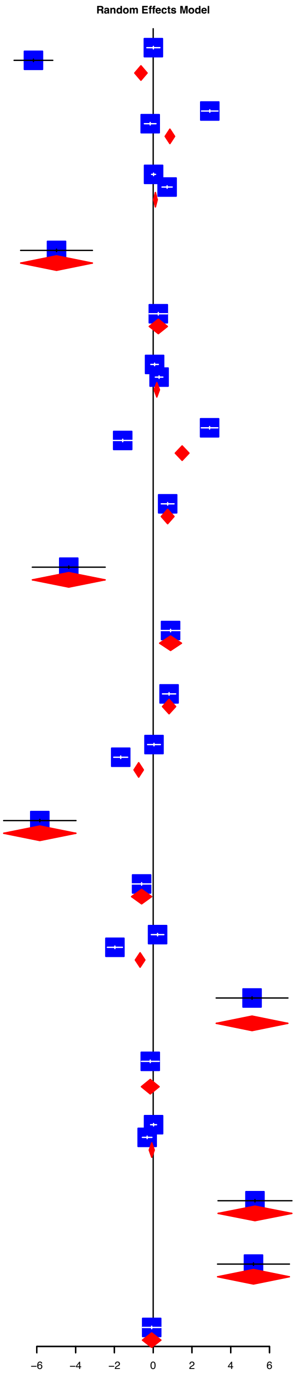

5.3 Adverse events

Comparison	Number of Studies	Direct Evidence	I2	OR	95%-CI
**Bisphosphonates:Calcitonin**					
Direct estimate	2	0.85	0%	0.52	[0.11; 2.49]
Indirect estimate				4.49	[0.10; 196.97]
Network estimate				0.71	[0.17; 3.03]
**Bisphosphonates:Calcium**					
Direct estimate	2	0.96	22%	2.44	[0.68; 8.71]
Indirect estimate				6.07	[0.01; 4272.37]
Network estimate				2.52	[0.72; 8.80]
**Bisphosphonates:Control**					
Direct estimate	3	0.75	10%	0.67	[0.28; 1.59]
Indirect estimate				0.71	[0.16; 3.17]
Network estimate				0.68	[0.32; 1.44]
**Bisphosphonates:Denosumab**					
Direct estimate	1	0.90		0.90	[0.32; 2.53]
Indirect estimate				1.36	[0.07; 28.35]
Network estimate				0.94	[0.35; 2.50]
**Bisphosphonates:Teriparatide**					
Direct estimate	0	0			
Indirect estimate				0.68	[0.03; 13.47]
Network estimate				0.68	[0.03; 13.47]
**Bisphosphonates:Vitamin_D**					
Direct estimate	3	0.54	56%	0.74	[0.23; 2.41]
Indirect estimate				0.70	[0.20; 2.48]
Network estimate				0.72	[0.30; 1.71]
**Calcitonin:Calcium**					
Direct estimate	1	0.70		2.36	[0.36; 15.45]
Indirect estimate				8.91	[0.52; 153.84]
Network estimate				3.53	[0.74; 16.94]
**Calcitonin:Control**					
Direct estimate	0	0			
Indirect estimate				0.95	[0.19; 4.85]
Network estimate				0.95	[0.19; 4.85]
**Calcitonin:Denosumab**					
Direct estimate	0	0			
Indirect estimate				1.32	[0.23; 7.56]
Network estimate				1.32	[0.23; 7.56]
**Calcitonin:Teriparatide**					
Direct estimate	0	0			
Indirect estimate				0.95	[0.03; 26.34]
Network estimate				0.95	[0.03; 26.34]
**Calcitonin:Vitamin_D**					
Direct estimate	0	0			
Indirect estimate				1.01	[0.19; 5.47]
Network estimate				1.01	[0.19; 5.47]
**Calcium:Control**					
Direct estimate	0	0			
Indirect estimate				0.27	[0.06; 1.16]
Network estimate				0.27	[0.06; 1.16]
**Calcium:Denosumab**					
Direct estimate	0	0			
Indirect estimate				0.37	[0.08; 1.83]
Network estimate				0.37	[0.08; 1.83]
**Calcium:Teriparatide**					
Direct estimate	0	0			
Indirect estimate				0.27	[0.01; 6.87]
Network estimate				0.27	[0.01; 6.87]
**Calcium:Vitamin_D**					
Direct estimate	0	0			
Indirect estimate				0.29	[0.06; 1.31]
Network estimate				0.29	[0.06; 1.31]
**Denosumab:Control**					
Direct estimate	1	0.76		0.67	[0.20; 2.25]
Indirect estimate				0.92	[0.11; 7.83]
Network estimate				0.72	[0.25; 2.07]
**Teriparatide:Control**					
Direct estimate	1	1.00		1.00	[0.06; 18.08]
Indirect estimate					
Network estimate				1.00	[0.06; 18.08]
**Vitamin_D:Control**					
Direct estimate	4	0.71	0%	0.95	[0.38; 2.41]
Indirect estimate				0.89	[0.21; 3.87]
Network estimate				0.94	[0.43; 2.05]
**Denosumab:Teriparatide**					
Direct estimate	0	0			
Indirect estimate				0.72	[0.03; 15.70]
Network estimate				0.72	[0.03; 15.70]
**Denosumab:Vitamin_D**					
Direct estimate	0	0			
Indirect estimate				0.77	[0.23; 2.56]
Network estimate				0.77	[0.23; 2.56]
**Teriparatide:Vitamin_D**					
Direct estimate	0	0			
Indirect estimate				1.07	[0.05; 21.42]
Network estimate				1.07	[0.05; 21.42]

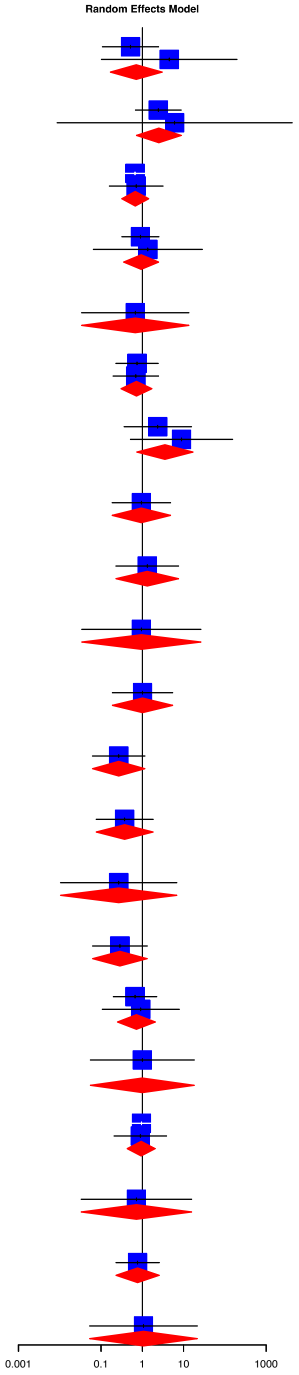

6 Funnel plots

6.1 Femoral neck bone mineral density

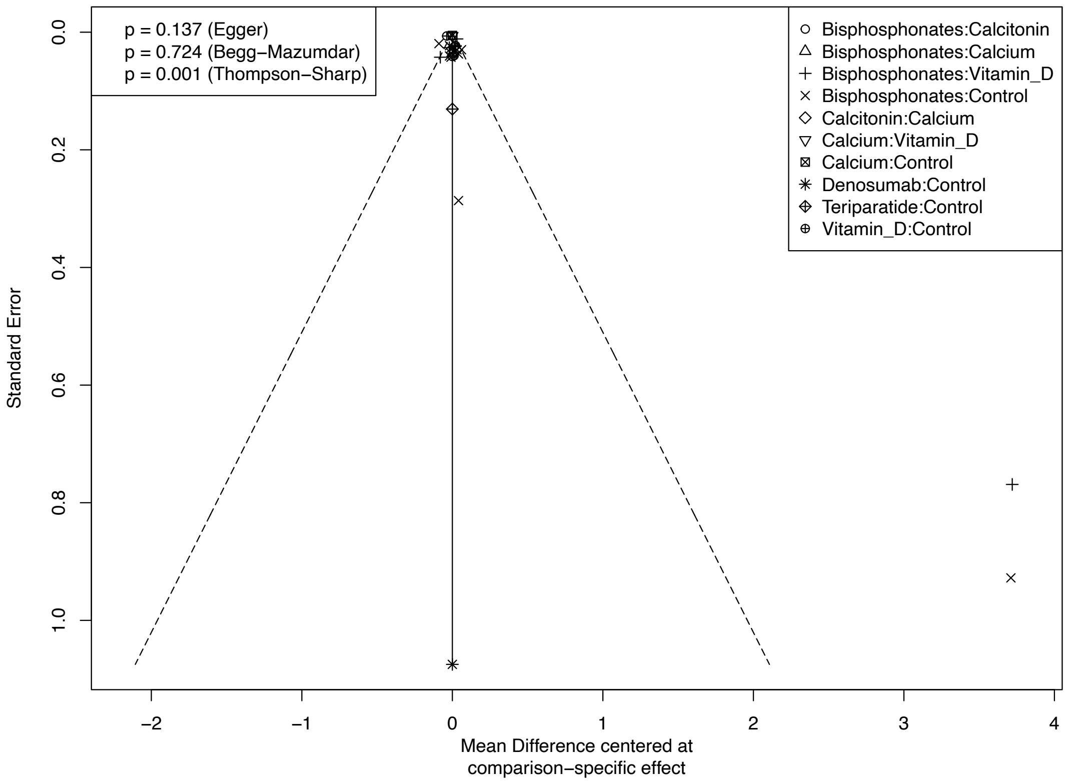

6.2 Lumbar spine bone mineral density

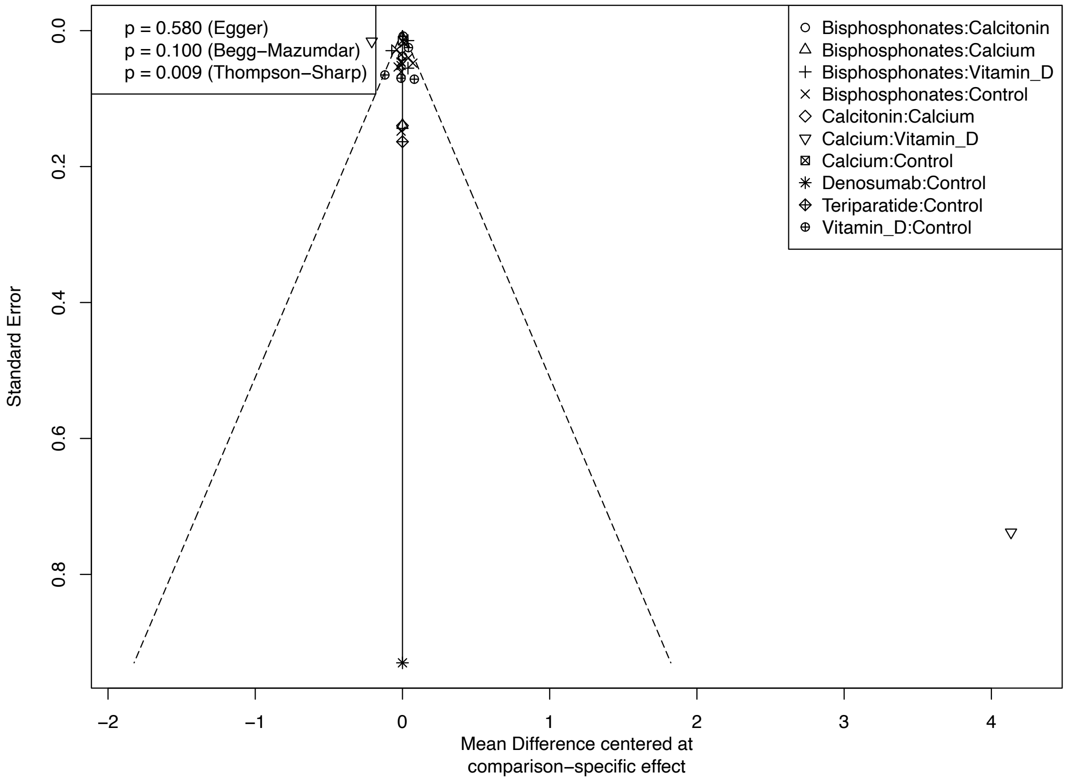

6.3 Adverse events

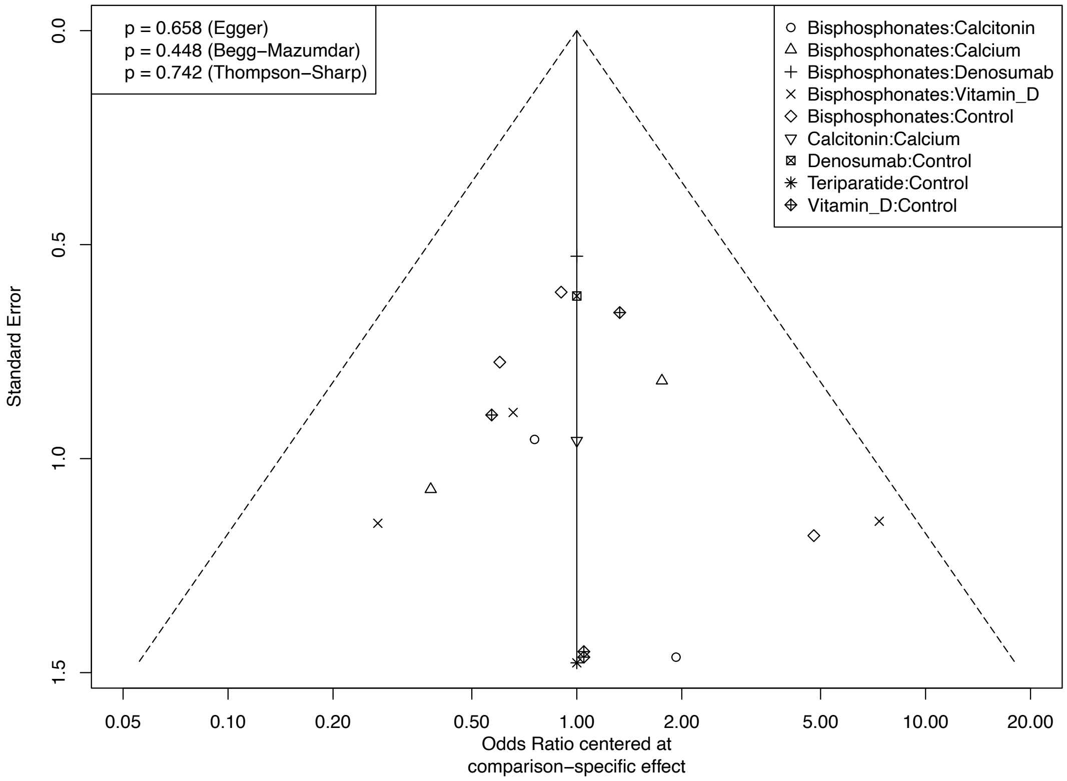

7 Results of assessments of the certainty of evidence for direct, indirect and network estimates

7.1 Femoral neck bone mineral density

Arm_1	Arm_2	No_of_study	Sample_size	I2	Direct_estimate	ROB	Inconsistency	Indirectness	Publication_bias	Direct_rating_without_imprecision	Indirect_estimate	First_order_loop_of_the_most_contribution	Certainty_of_evidence_for_arm 1	Certainty_of_evidence_for_arm 2	Intransitivity	Indirect_rating_without_imprecision	Network_meta_analysis	Higher_rating_of_direct_and_indirect_without_imprecision	Incoherence	Imprecision	Final_rating	Final_rating_reason	Evidence_type_for_final_rating
Bisphosphonates	Calcitonin	1	31	.	0.05 [-0.10; 0.20]	Not serious	Not serious	Not serious	Undetected	High	0.45 [0.12; 0.79]	Bisphosphonates - Calcium; Calcium - Calcitonin	High	High	Not serious	High	0.12 [-0.02; 0.25]	High	Serious	Serious	Moderate	Direct estimate was used because incoherence was significant and direct evidence had higher or equal certainty. Imprecision was rated as Serious.	direct
Bisphosphonates	Calcium	1	31	.	-0.15 [-0.30; -0.00]	Not serious	Not serious	Not serious	Undetected	High	0.05 [-0.06; 0.17]	Bisphosphonates - Control; Control - Calcium	Very low	Moderate	Not serious	Very low	-0.03 [-0.12; 0.06]	High	Serious	Serious	Moderate	Direct estimate was used because incoherence was significant and direct evidence had higher or equal certainty. Imprecision was rated as Serious.	direct
Bisphosphonates	Control	10	436	78.5%	0.04 [-0.01; 0.09]	Serious	Serious	Not serious	Serious	Very low	0.06 [-0.04; 0.16]	Bisphosphonates - Vitamin_D; Vitamin_D - Control	Low	Moderate	Not serious	Low	0.04 [0.00; 0.09]	Low	Not serious	Not serious	Low	NMA estimate was used because direct and indirect evidence were consistent. Imprecision was rated as Not serious.	network
Bisphosphonates	Denosumab	0	.	.	.						-1.06 [-3.17; 1.05]	Bisphosphonates - Control; Control - Denosumab	Very low	Moderate	Not serious	Very low	-1.06 [-3.17; 1.05]	Very low	Not serious	Serious	Very low	Indirect estimate was used because no direct evidence was available. Imprecision was rated as Serious.	indirect
Bisphosphonates	Teriparatide	0	.	.	.						0.01 [-0.28; 0.30]	Bisphosphonates - Control; Control - Teriparatide	Very low	High	Not serious	Very low	0.01 [-0.28; 0.30]	Very low	Not serious	Serious	Very low	Indirect estimate was used because no direct evidence was available. Imprecision was rated as Serious.	indirect
Bisphosphonates	Vitamin_D	5	290	87.4%	0.03 [-0.04; 0.10]	Serious	Serious	Not serious	Undetected	Low	-0.05 [-0.14; 0.03]	Bisphosphonates - Control; Control - Vitamin_D	Very low	Moderate	Not serious	Very low	-0.00 [-0.06; 0.05]	Low	Not serious	Serious	Very low	NMA estimate was used because direct and indirect evidence were consistent. Imprecision was rated as Serious.	network
Calcitonin	Calcium	1	30	.	-0.20 [-0.34; -0.06]	Not serious	Not serious	Not serious	Undetected	High	0.24 [-0.14; 0.61]	Calcitonin - Bisphosphonates; Bisphosphonates - Calcium	High	High	Not serious	High	-0.14 [-0.28; -0.01]	High	Serious	Serious	Moderate	Direct estimate was used because incoherence was significant and direct evidence had higher or equal certainty. Imprecision was rated as Serious.	direct
Calcitonin	Control	0	.	.	.						-0.07 [-0.21; 0.07]	Calcitonin - Bisphosphonates; Bisphosphonates - Control	High	Very low	Not serious	Very low	-0.07 [-0.21; 0.07]	Very low	Not serious	Serious	Very low	Indirect estimate was used because no direct evidence was available. Imprecision was rated as Serious.	indirect
Calcitonin	Denosumab	0	.	.	.						-1.17 [-3.29; 0.94]		Unrated	Moderate	Not serious	Moderate	-1.17 [-3.29; 0.94]	Moderate	Not serious	Serious	Low	Indirect estimate was used because no direct evidence was available. Imprecision was rated as Serious.	indirect

Calcitonin	Teriparatide	0	.	.	.						-0.10 [-0.42; 0.22]		Unrated	High	Not serious	High	-0.10 [-0.42; 0.22]	High	Not serious	Serious	Moderate	Indirect estimate was used because no direct evidence was available. Imprecision was rated as Serious.	indirect
Calcitonin	Vitamin_D	0	.	.	.						-0.12 [-0.26; 0.02]	Calcitonin - Bisphosphonates; Bisphosphonates - Vitamin_D	High	Low	Not serious	Low	-0.12 [-0.26; 0.02]	Low	Not serious	Serious	Very low	Indirect estimate was used because no direct evidence was available. Imprecision was rated as Serious.	indirect
Calcium	Control	1	25	.	-0.01 [-0.13; 0.12]	Serious	Not serious	Not serious	Undetected	Moderate	0.16 [0.02; 0.29]	Calcium - Bisphosphonates; Bisphosphonates - Control	High	Very low	Not serious	Very low	0.07 [-0.02; 0.16]	Moderate	Not serious	Serious	Low	NMA estimate was used because direct and indirect evidence were consistent. Imprecision was rated as Serious.	network
Calcium	Denosumab	0	.	.	.						-1.03 [-3.14; 1.08]	Calcium - Control; Control - Denosumab	Moderate	Moderate	Not serious	Moderate	-1.03 [-3.14; 1.08]	Moderate	Not serious	Serious	Low	Indirect estimate was used because no direct evidence was available. Imprecision was rated as Serious.	indirect
Calcium	Teriparatide	0	.	.	.						0.04 [-0.26; 0.34]	Calcium - Control; Control - Teriparatide	Moderate	High	Not serious	Moderate	0.04 [-0.26; 0.34]	Moderate	Not serious	Serious	Low	Indirect estimate was used because no direct evidence was available. Imprecision was rated as Serious.	indirect
Calcium	Vitamin_D	1	24	.	-0.04 [-0.16; 0.09]	Serious	Not serious	Not serious	Undetected	Moderate	0.10 [-0.04; 0.24]	Calcium - Bisphosphonates; Bisphosphonates - Vitamin_D	High	Low	Not serious	Low	0.03 [-0.07; 0.12]	Moderate	Not serious	Serious	Low	NMA estimate was used because direct and indirect evidence were consistent. Imprecision was rated as Serious.	network
Denosumab	Control	1	90	.	1.10 [-1.01; 3.21]	Serious	Not serious	Not serious	Undetected	Moderate	.						1.10 [-1.01; 3.21]	Moderate	Not serious	Serious	Low	Direct estimate was used because no indirect evidence was available. Imprecision was rated as Serious.	direct
Teriparatide	Control	1	24	.	0.03 [-0.26; 0.32]	Not serious	Not serious	Not serious	Undetected	High	.						0.03 [-0.26; 0.32]	High	Not serious	Serious	Moderate	Direct estimate was used because no indirect evidence was available. Imprecision was rated as Serious.	direct
Vitamin_D	Control	4	156	70.8%	0.07 [0.00; 0.14]	Serious	Not serious	Not serious	Undetected	Moderate	0.00 [-0.09; 0.09]	Vitamin_D - Bisphosphonates; Bisphosphonates - Control	Low	Very low	Not serious	Very low	0.05 [-0.01; 0.10]	Moderate	Not serious	Serious	Low	NMA estimate was used because direct and indirect evidence were consistent. Imprecision was rated as Serious.	network
Denosumab	Teriparatide	0	.	.	.						1.07 [-1.06; 3.20]	Denosumab - Control; Control - Teriparatide	Moderate	High	Not serious	Moderate	1.07 [-1.06; 3.20]	Moderate	Not serious	Serious	Low	Indirect estimate was used because no direct evidence was available. Imprecision was rated as Serious.	indirect
Denosumab	Vitamin_D	0	.	.	.						1.05 [-1.06; 3.17]	Denosumab - Control; Control - Vitamin_D	Moderate	Moderate	Not serious	Moderate	1.05 [-1.06; 3.17]	Moderate	Not serious	Serious	Low	Indirect estimate was used because no direct evidence was available. Imprecision was rated as Serious.	indirect
Teriparatide	Vitamin_D	0	.	.	.						-0.02 [-0.31; 0.28]	Teriparatide - Control; Control - Vitamin_D	High	Moderate	Not serious	Moderate	-0.02 [-0.31; 0.28]	Moderate	Not serious	Serious	Low	Indirect estimate was used because no direct evidence was available. Imprecision was rated as Serious.	indirect

7.2 Lumbar spine bone mineral density

Arm_1	Arm_2	No_of_study	Sample_size	I2	Direct_estimate	ROB	Inconsistency	Indirectness	Publication_bias	Direct_rating_without_imprecision	Indirect_estimate	First_order_loop_of_the_most_contribution	Certainty_of_evidence_for_arm1	Certainty_of_evidence_for_arm2	Intransitivity	Indirect_rating_without_imprecision	Network_meta_analysis	Higher_rating_of_direct_and_indirect_without_imprecision	Incoherence	Imprecision	Final_rating	Final_rating_reason	Evidence_type_for_final_rating
Bisphosphonates	Calcitonin	1	31	.	0.01 [-0.33; 0.35]	Not serious	Not serious	Not serious	Undetected	High	-6.18 [-7.18; -5.18]	Bisphosphonates - Calcium; Calcium - Calcitonin	High	High	Not serious	High	-0.63 [-0.95; -0.31]	High	Serious	Serious	Moderate	Direct estimate was used because incoherence was significant and direct evidence had higher or equal certainty. Imprecision was rated as Serious.	direct
Bisphosphonates	Calcium	1	31	.	2.92 [2.49; 3.35]	Not serious	Not serious	Not serious	Undetected	High	-0.15 [-0.46; 0.15]	Bisphosphonates - Control; Control - Calcium	Low	Moderate	Not serious	Low	0.86 [0.62; 1.11]	High	Serious	Serious	Moderate	Direct estimate was used because incoherence was significant and direct evidence had higher or equal certainty. Imprecision was rated as Serious.	direct
Bisphosphonates	Control	11	476	0.0%	0.02 [-0.09; 0.12]	Serious	Not serious	Not serious	Serious	Low	0.72 [0.45; 0.98]	Bisphosphonates - Vitamin_D; Vitamin_D - Control	Moderate	Moderate	Not serious	Moderate	0.12 [0.02; 0.21]	Moderate	Serious	Serious	Low	Indirect estimate was used because incoherence was significant and indirect evidence had higher certainty. Imprecision was rated as Serious.	indirect
Bisphosphonates	Denosumab	0	.	.	.						-4.98 [-6.84; -3.13]	Bisphosphonates - Control; Control - Denosumab	Low	Moderate	Not serious	Low	-4.98 [-6.84; -3.13]	Low	Not serious	Serious	Very low	Indirect estimate was used because no direct evidence was available. Imprecision was rated as Serious.	indirect
Bisphosphonates	Teriparatide	0	.	.	.						0.27 [-0.21; 0.74]	Bisphosphonates - Control; Control - Teriparatide	Low	High	Not serious	Low	0.27 [-0.21; 0.74]	Low	Not serious	Serious	Very low	Indirect estimate was used because no direct evidence was available. Imprecision was rated as Serious.	indirect
Bisphosphonates	Vitamin_D	3	206	82.4%	0.07 [-0.12; 0.27]	Serious	Not serious	Not serious	Undetected	Moderate	0.31 [0.11; 0.50]	Bisphosphonates - Control; Control - Vitamin_D	Low	Moderate	Not serious	Low	0.19 [0.05; 0.33]	Moderate	Not serious	Serious	Low	NMA estimate was used because direct and indirect evidence were consistent. Imprecision was rated as Serious.	network
Calcitonin	Calcium	1	30	.	2.91 [2.48; 3.34]	Not serious	Not serious	Not serious	Undetected	High	-1.57 [-2.21; -0.94]	Calcitonin - Bisphosphonates; Bisphosphonates - Calcium	High	High	Not serious	High	1.49 [1.14; 1.85]	High	Serious	Serious	Moderate	Direct estimate was used because incoherence was significant and direct evidence had higher or equal certainty. Imprecision was rated as Serious.	direct
Calcitonin	Control	0	.	.	.						0.75 [0.41; 1.08]	Calcitonin - Bisphosphonates; Bisphosphonates - Control	High	Low	Not serious	Low	0.75 [0.41; 1.08]	Low	Not serious	Serious	Very low	Indirect estimate was used because no direct evidence was available. Imprecision was rated as Serious.	indirect
Calcitonin	Denosumab	0	.	.	.						-4.35 [-6.24; -2.47]		Unrated	Moderate	Not serious	Moderate	-4.35 [-6.24; -2.47]	Moderate	Not serious	Serious	Low	Indirect estimate was used because no direct evidence was available. Imprecision was rated as Serious.	indirect
Calcitonin	Teriparatide	0	.	.	.						0.90 [0.33; 1.46]		Unrated	High	Not serious	High	0.90 [0.33; 1.46]	High	Not serious	Serious	Moderate	Indirect estimate was used because no direct evidence was available. Imprecision was rated as Serious.	indirect

Calcitonin	Vitamin_D	0	.	.	.						0.82 [0.48; 1.16]	Calcitonin - Bisphosphonates; Bisphosphonates - Vitamin_D	High	Moderate	Not serious	Moderate	0.82 [0.48; 1.16]	Moderate	Not serious	Serious	Low	Indirect estimate was used because no direct evidence was available. Imprecision was rated as Serious.	indirect
Calcium	Control	1	25	.	0.04 [-0.29; 0.38]	Serious	Not serious	Not serious	Undetected	Moderate	-1.67 [-2.04; -1.31]	Calcium - Bisphosphonates; Bisphosphonates - Control	High	Low	Not serious	Low	-0.75 [-0.99; -0.50]	Moderate	Serious	Serious	Low	Direct estimate was used because incoherence was significant and direct evidence had higher or equal certainty. Imprecision was rated as Serious.	direct
Calcium	Denosumab	0	.	.	.						-5.85 [-7.72; -3.98]	Calcium - Control; Control - Denosumab	Moderate	Moderate	Not serious	Moderate	-5.85 [-7.72; -3.98]	Moderate	Not serious	Serious	Low	Indirect estimate was used because no direct evidence was available. Imprecision was rated as Serious.	indirect
Calcium	Teriparatide	0	.	.	.						-0.60 [-1.12; -0.07]	Calcium - Control; Control - Teriparatide	Moderate	High	Not serious	Moderate	-0.60 [-1.12; -0.07]	Moderate	Not serious	Serious	Low	Indirect estimate was used because no direct evidence was available. Imprecision was rated as Serious.	indirect
Calcium	Vitamin_D	2	62	97.1%	0.23 [-0.10; 0.55]	Serious	Very serious	Not serious	Undetected	Very low	-1.97 [-2.36; -1.58]	Calcium - Control; Control - Vitamin_D	Moderate	Moderate	Not serious	Moderate	-0.67 [-0.92; -0.42]	Moderate	Serious	Serious	Low	Indirect estimate was used because incoherence was significant and indirect evidence had higher certainty. Imprecision was rated as Serious.	indirect
Denosumab	Control	1	90	.	5.10 [3.25; 6.95]	Serious	Not serious	Not serious	Undetected	Moderate	.						5.10 [3.25; 6.95]	Moderate	Not serious	Serious	Low	Direct estimate was used because no indirect evidence was available. Imprecision was rated as Serious.	direct
Teriparatide	Control	1	24	.	-0.15 [-0.61; 0.31]	Not serious	Not serious	Not serious	Undetected	High	.						-0.15 [-0.61; 0.31]	High	Not serious	Serious	Moderate	Direct estimate was used because no indirect evidence was available. Imprecision was rated as Serious.	direct
Vitamin_D	Control	5	343	41.0%	0.02 [-0.14; 0.18]	Serious	Not serious	Not serious	Undetected	Moderate	-0.31 [-0.56; -0.06]	Vitamin_D - Bisphosphonates; Bisphosphonates - Control	Moderate	Low	Not serious	Low	-0.07 [-0.21; 0.06]	Moderate	Serious	Serious	Low	Direct estimate was used because incoherence was significant and direct evidence had higher or equal certainty. Imprecision was rated as Serious.	direct
Denosumab	Teriparatide	0	.	.	.						5.25 [3.34; 7.16]	Denosumab - Control; Control - Teriparatide	Moderate	High	Not serious	Moderate	5.25 [3.34; 7.16]	Moderate	Not serious	Serious	Low	Indirect estimate was used because no direct evidence was available. Imprecision was rated as Serious.	indirect
Denosumab	Vitamin_D	0	.	.	.						5.17 [3.32; 7.03]	Denosumab - Control; Control - Vitamin_D	Moderate	Moderate	Not serious	Moderate	5.17 [3.32; 7.03]	Moderate	Not serious	Serious	Low	Indirect estimate was used because no direct evidence was available. Imprecision was rated as Serious.	indirect
Teriparatide	Vitamin_D	0	.	.	.						-0.08 [-0.56; 0.40]	Teriparatide - Control; Control - Vitamin_D	High	Moderate	Not serious	Moderate	-0.08 [-0.56; 0.40]	Moderate	Not serious	Serious	Low	Indirect estimate was used because no direct evidence was available. Imprecision was rated as Serious.	indirect

7.3 Adverse events

Arm_1	Arm_2	No_of_study	Sample_size	I2	Direct_estimate	ROB	Inconsistency	Indirectness	Publication_bias	Direct_rating_without_imprecision	Indirect_estimate	First_order_loop_of_the_most_contribution	Certainty_of_evidence_for_arm1	Certainty_of_evidence_for_arm2	Intransitivity	Indirect_rating_without_imprecision	Network_meta_analysis	Higher_rating_of_direct_and_indirect_without_imprecision	Incoherence	Imprecision	Final_rating	Final_rating_reason	Evidence_type_for_final_rating
Bisphosphonates	Calcitonin	2	61	0.0%	0.52 [0.11; 2.49]	Serious	Not serious	Not serious	Undetected	Moderate	4.49 [0.10; 196.97]	Bisphosphonates - Calcium; Calcium - Calcitonin	Moderate	Moderate	Not serious	Moderate	0.71 [0.17; 3.03]	Moderate	Not serious	Serious	Low	NMA estimate was used because direct and indirect evidence were consistent. Imprecision was rated as Serious.	network
Bisphosphonates	Calcium	2	132	22.0%	2.44 [0.68; 8.71]	Serious	Not serious	Not serious	Undetected	Moderate	6.07 [0.01; 4272.37]	Bisphosphonates - Calcitonin; Calcitonin - Calcium	Moderate	Moderate	Not serious	Moderate	2.52 [0.72; 8.80]	Moderate	Not serious	Serious	Low	NMA estimate was used because direct and indirect evidence were consistent. Imprecision was rated as Serious.	network
Bisphosphonates	Control	3	181	9.9%	0.67 [0.28; 1.59]	Serious	Not serious	Not serious	Undetected	Moderate	0.71 [0.16; 3.17]	Bisphosphonates - Vitamin_D; Vitamin_D - Control	Moderate	Moderate	Not serious	Moderate	0.68 [0.32; 1.44]	Moderate	Not serious	Serious	Low	NMA estimate was used because direct and indirect evidence were consistent. Imprecision was rated as Serious.	network
Bisphosphonates	Denosumab	1	79	.	0.90 [0.32; 2.53]	Serious	Not serious	Not serious	Undetected	Moderate	1.36 [0.07; 28.35]	Bisphosphonates - Control; Control - Denosumab	Moderate	Moderate	Not serious	Moderate	0.94 [0.35; 2.50]	Moderate	Not serious	Serious	Low	NMA estimate was used because direct and indirect evidence were consistent. Imprecision was rated as Serious.	network
Bisphosphonates	Teriparatide	0	.	.	.						0.68 [0.03; 13.47]	Bisphosphonates - Control; Control - Teriparatide	Moderate	High	Not serious	Moderate	0.68 [0.03; 13.47]	Moderate	Not serious	Serious	Low	Indirect estimate was used because no direct evidence was available. Imprecision was rated as Serious.	indirect
Bisphosphonates	Vitamin_D	3	221	56.1%	0.74 [0.23; 2.41]	Serious	Not serious	Not serious	Undetected	Moderate	0.70 [0.20; 2.48]	Bisphosphonates - Control; Control - Vitamin_D	Moderate	Moderate	Not serious	Moderate	0.72 [0.30; 1.71]	Moderate	Not serious	Serious	Low	NMA estimate was used because direct and indirect evidence were consistent. Imprecision was rated as Serious.	network
Calcitonin	Calcium	1	30	.	2.36 [0.36; 15.45]	Serious	Not serious	Not serious	Undetected	Moderate	8.91 [0.52; 153.84]	Calcitonin - Bisphosphonates; Bisphosphonates - Calcium	Moderate	Moderate	Not serious	Moderate	3.53 [0.74; 16.94]	Moderate	Not serious	Serious	Low	NMA estimate was used because direct and indirect evidence were consistent. Imprecision was rated as Serious.	network
Calcitonin	Control	0	.	.	.						0.95 [0.19; 4.85]	Calcitonin - Bisphosphonates; Bisphosphonates - Control	Moderate	Moderate	Not serious	Moderate	0.95 [0.19; 4.85]	Moderate	Not serious	Serious	Low	Indirect estimate was used because no direct evidence was available. Imprecision was rated as Serious.	indirect
Calcitonin	Denosumab	0	.	.	.						1.32 [0.23; 7.56]	Calcitonin - Bisphosphonates; Bisphosphonates - Denosumab	Moderate	Moderate	Not serious	Moderate	1.32 [0.23; 7.56]	Moderate	Not serious	Serious	Low	Indirect estimate was used because no direct evidence was available. Imprecision was rated as Serious.	indirect
Calcitonin	Teriparatide	0	.	.	.						0.95 [0.03; 26.34]		Unrated	High	Not serious	High	0.95 [0.03; 26.34]	High	Not serious	Serious	Moderate	Indirect estimate was used because no direct evidence was available. Imprecision was rated as Serious.	indirect

Calcitonin	Vitamin_D	0	.	.	.						1.01 [0.19; 5.47]	Calcitonin - Bisphosphonates; Bisphosphonates - Vitamin_D	Moderate	Moderate	Not serious	Moderate	1.01 [0.19; 5.47]	Moderate	Not serious	Serious	Low	Indirect estimate was used because no direct evidence was available. Imprecision was rated as Serious.	indirect
Calcium	Control	0	.	.	.						0.27 [0.06; 1.16]	Calcium - Bisphosphonates; Bisphosphonates - Control	Moderate	Moderate	Not serious	Moderate	0.27 [0.06; 1.16]	Moderate	Not serious	Serious	Low	Indirect estimate was used because no direct evidence was available. Imprecision was rated as Serious.	indirect
Calcium	Denosumab	0	.	.	.						0.37 [0.08; 1.83]	Calcium - Bisphosphonates; Bisphosphonates - Denosumab	Moderate	Moderate	Not serious	Moderate	0.37 [0.08; 1.83]	Moderate	Not serious	Serious	Low	Indirect estimate was used because no direct evidence was available. Imprecision was rated as Serious.	indirect
Calcium	Teriparatide	0	.	.	.						0.27 [0.01; 6.87]		Unrated	High	Not serious	High	0.27 [0.01; 6.87]	High	Not serious	Serious	Moderate	Indirect estimate was used because no direct evidence was available. Imprecision was rated as Serious.	indirect
Calcium	Vitamin_D	0	.	.	.						0.29 [0.06; 1.31]	Calcium - Bisphosphonates; Bisphosphonates - Vitamin_D	Moderate	Moderate	Not serious	Moderate	0.29 [0.06; 1.31]	Moderate	Not serious	Serious	Low	Indirect estimate was used because no direct evidence was available. Imprecision was rated as Serious.	indirect
Denosumab	Control	1	69	.	0.67 [0.20; 2.25]	Serious	Not serious	Not serious	Undetected	Moderate	0.92 [0.11; 7.83]	Denosumab - Bisphosphonates; Bisphosphonates - Control	Moderate	Moderate	Not serious	Moderate	0.72 [0.25; 2.07]	Moderate	Not serious	Serious	Low	NMA estimate was used because direct and indirect evidence were consistent. Imprecision was rated as Serious.	network
Teriparatide	Control	1	24	.	1.00 [0.06; 18.08]	Not serious	Not serious	Not serious	Undetected	High	.						1.00 [0.06; 18.08]	High	Not serious	Serious	Moderate	Direct estimate was used because no indirect evidence was available. Imprecision was rated as Serious.	direct
Vitamin_D	Control	4	258	0.0%	0.95 [0.38; 2.41]	Serious	Not serious	Not serious	Undetected	Moderate	0.89 [0.21; 3.87]	Vitamin_D - Bisphosphonates; Bisphosphonates - Control	Moderate	Moderate	Not serious	Moderate	0.94 [0.43; 2.05]	Moderate	Not serious	Serious	Low	NMA estimate was used because direct and indirect evidence were consistent. Imprecision was rated as Serious.	network
Denosumab	Teriparatide	0	.	.	.						0.72 [0.03; 15.70]	Denosumab - Control; Control - Teriparatide	Moderate	High	Not serious	Moderate	0.72 [0.03; 15.70]	Moderate	Not serious	Serious	Low	Indirect estimate was used because no direct evidence was available. Imprecision was rated as Serious.	indirect
Denosumab	Vitamin_D	0	.	.	.						0.77 [0.23; 2.56]	Denosumab - Control; Control - Vitamin_D	Moderate	Moderate	Not serious	Moderate	0.77 [0.23; 2.56]	Moderate	Not serious	Serious	Low	Indirect estimate was used because no direct evidence was available. Imprecision was rated as Serious.	indirect
Teriparatide	Vitamin_D	0	.	.	.						1.07 [0.05; 21.42]	Teriparatide - Control; Control - Vitamin_D	High	Moderate	Not serious	Moderate	1.07 [0.05; 21.42]	Moderate	Not serious	Serious	Low	Indirect estimate was used because no direct evidence was available. Imprecision was rated as Serious.	indirect
